# Antimony(III) and Bismuth(III) Halide Complexes Containing Thiazole-and Thiadiazole-Based Mercapto Ligands: Synthesis, Structural Characterization, and Biological Activity

**DOI:** 10.3390/molecules31203783

**Published:** 2026-10-21

**Authors:** Ibrahim I. Ozturk, Cemile Albayrak, Anita M. Grześkiewicz, Maciej Kubicki, Christina N. Banti, Sotiris K. Hadjikakou

**Affiliations:** 1 Section of Inorganic Chemistry, Department of Chemistry, Tekirdag Namık Kemal University, 59030 Tekirdag, Türkiye; 2 Department of Chemistry, A. Mickiewicz University, ul. Uniwersytetu Poznanskiego 8, 61-614 Poznan, Poland; 3 Biological Inorganic Chemistry Laboratory, Department of Chemistry, University of Ioannina, 45110 Ioannina, Greece

**Keywords:** antimony(III) complexes, bismuth(III) complexes, thioamide ligands, supramolecular assembly, antiproliferative activity, antibacterial activity

## Abstract

Sulfur-rich heterocyclic ligands are widely employed as building blocks for biologically active main-group metal complexes owing to their versatile coordination behavior. In this study, four new antimony(III) and bismuth(III) halide complexes based on 2-mercapto-4-methylthiazole and 2-mercapto-5-methyl-1,3,4-thiadiazole ligands were synthesized, spectroscopically characterized, and evaluated for their biological activity. The crystal structures of complexes **1**, **3**, and **4** were determined by single-crystal X-ray diffraction, revealing distorted square-pyramidal coordination geometries with the ligands coordinated in their neutral thione form through the sulfur donor atom. Single-crystal X-ray diffraction analyses reveal distinct solid-state arrangements: complex **1** is monomeric, complex **3** forms a chloride-bridged polymer, and complex **4** forms bromide-bridged dimers. Time-dependent molar conductivity and UV–Vis measurements showed no substantial changes under the investigated conditions. The antimony(III) complexes exhibited antiproliferative activity against HeLa cells, whereas the bismuth(III) analogues were inactive within the investigated concentration range. The complexes also showed modest antibacterial effects in the disc-diffusion screening assay, with the most pronounced inhibition-zone responses observed against *E. coli*. Overall, the biological results reveal differences in activity among the investigated Sb(III) and Bi(III) complexes under the tested conditions.

## 1. Introduction

Thiazole and thiadiazole derivatives have been extensively studied due to their diverse pharmacological properties and their roles in various biological systems [1,2,3,4,5,6,7]. These compounds form the core structures of numerous therapeutically relevant agents exhibiting antimicrobial, antifungal, anti-inflammatory, anticancer, and antiretroviral activities [8,9,10,11,12,13,14,15,16,17].

In coordination chemistry, N-heterocyclic thiones containing a thioamide group represent versatile ligands, as they can coordinate through both the ring nitrogen and exocyclic sulfur atoms, enabling diverse coordination modes [18,19,20,21,22,23,24]. In line with the HSAB (Pearson’s hard–soft acid–base) principle, these ligands readily form complexes with both transition and main-group metal ions [25,26]. Their structural flexibility allows them to act as monodentate, chelating, or bridging ligands, facilitating the formation of multinuclear assemblies and extended supramolecular architectures [27,28,29,30,31,32,33]. A key feature of these compounds is their prototropic tautomerism between thiol and thione forms. In particular, derivatives such as 2-mercapto-4-methylthiazole (Hmmthz) and 2-mercapto-5-methyl-1,3,4-thiadiazole (Hmmthd) predominantly exist in the thione form in the solid state and in selected solution environments [32,33,34,35,36,37,38,39,40,41,42,43,44].

Recent reports demonstrate that organosulfur ligands can enhance the antimicrobial and anticancer properties of Sb(III) and Bi(III) complexes. Several structurally characterized complexes have been shown to exhibit selective cytotoxicity toward cancer cell lines and activity against both Gram-positive and Gram-negative bacteria [45,46,47,48,49,50,51,52]. Despite the extensive literature on sulfur-rich heterocycles, studies investigating both Hmmthz and Hmmthd within the same Sb(III)/Bi(III) halide framework remain limited [38,53,54]. Electronic differences between thiazole and thiadiazole rings—such as nitrogen distribution, sulfur basicity, and aromatic stabilization—may influence coordination behavior, supramolecular organization, and biological activity [55,56,57].

In this study, new Sb(III) and Bi(III) halide complexes derived from Hmmthz and Hmmthd ligands were synthesized and characterized by spectroscopic methods. The crystal structures of complexes **1**, **3**, and **4** were further elucidated by single-crystal X-ray diffraction. Their antibacterial activities and antiproliferative effects against HeLa cells were also evaluated as an initial assessment of their biological properties. This study provides structural and preliminary biological information on thioamide-based main-group metal complexes and enables a cautious comparison of the biological responses observed for the investigated compounds.

## 2. Results and Discussion

### 2.1. Synthesis

**Scheme 1:**
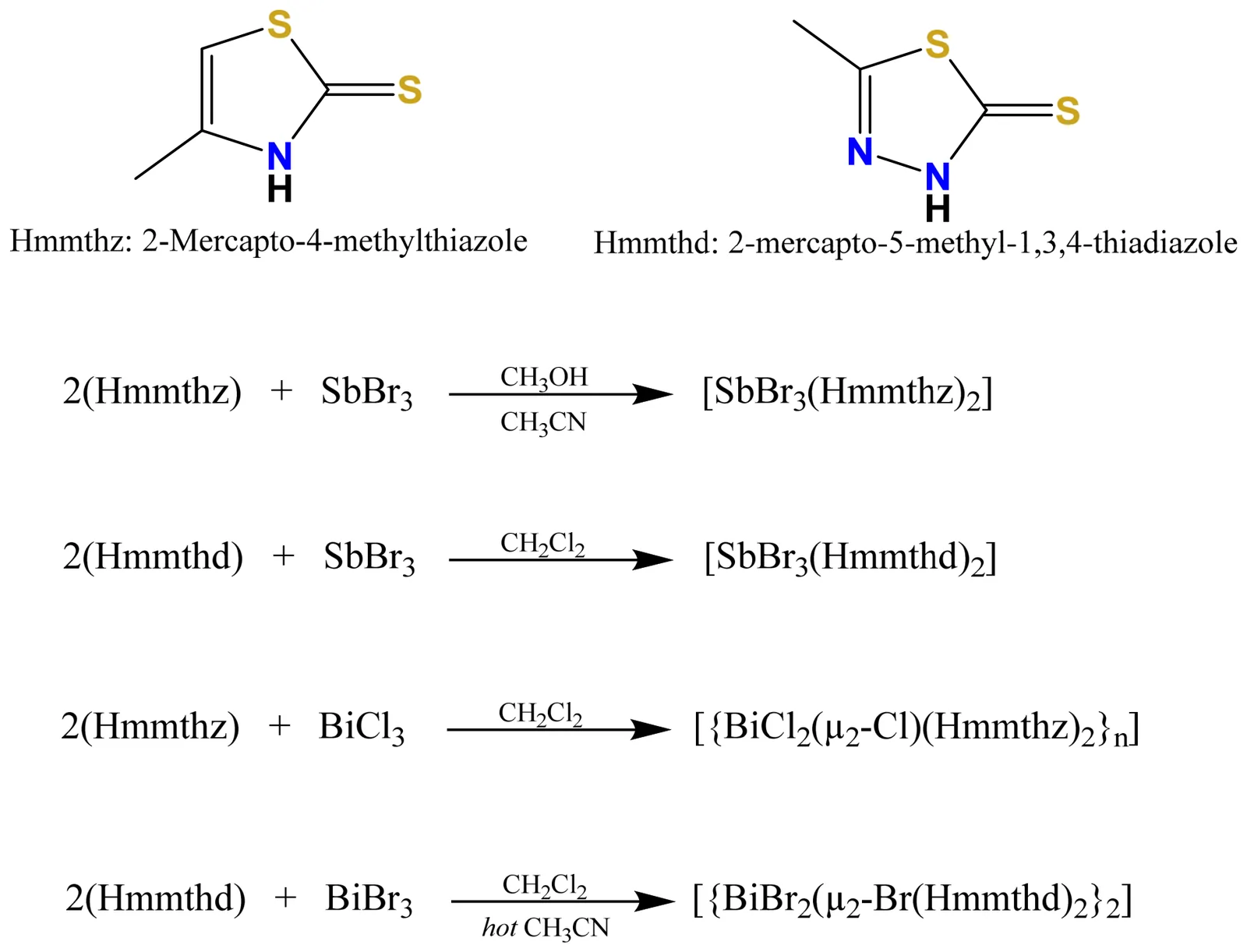
Proposed synthetic route for complexes **1**–**4** obtained from the reaction of SbX_3_ or BiX_3_ (X = halide) with mercapto heterocyclic ligands in common organic solvents.

Two antimony(III) and two bismuth(III) halide complexes were synthesized by reacting one equivalent of Sb(III) or Bi(III) salts with two equivalents of mercapto-functionalized heterocyclic ligands (Scheme 1). Complexes **1**–**3** were isolated from clear filtrates by slow evaporation at ambient temperature, whereas complex **4** was obtained via recrystallization from hot acetonitrile after filtration and drying of the initially formed precipitate. Complexes **1**, **2**, and **4** were obtained as yellow crystals, while complex **3** was isolated as orange crystals. The products were obtained in high yields (78–86%) and were stable under ambient laboratory conditions. All complexes are readily soluble in common polar organic solvents such as methanol, ethanol, acetone, acetonitrile, chloroform, dimethylformamide, and dimethyl sulfoxide. BiCl_3_ and BiBr_3_ were used as Bi(III) starting materials to obtain chloride- and bromide-containing complexes and to explore the coordination chemistry of these two halides with the ligands used in this study. The resulting compounds do not constitute a strictly matched series for isolating the effect of halide identity, since the Bi(III) complexes also differ in ligand identity.

The coordination behavior of the ligands indicates that the mercapto-functionalized heterocycles bind to the metal centers in their neutral thione form via the sulfur donor atom in a monodentate manner. The persistence of N–H stretching vibrations (see spectroscopy section) suggests that no deprotonation occurs during coordination. This behavior is consistent with the soft Lewis acid character of Sb(III) and Bi(III) ions, which exhibit a strong affinity toward sulfur donors. The observed 2:1 ligand-to-metal stoichiometry is consistent with the formation of five-coordinate metal centers. The crystal structures of complexes **1**, **3**, and **4** confirm distorted square-pyramidal coordination geometries.

### 2.2. Molar Conductivity and Electrolytic Behavior of the Complexes

The molar conductivities of complexes **1**–**4** were measured in 10^−3^ M solutions at 25 °C in DMF and DMSO. In DMF, the conductivity values ranged from 5.42 ± 0.21 to 10.12 ± 0.74 Ω^−1^ cm^2^ mol^−1^, whereas in DMSO they ranged from 11.25 ± 0.08 to 19.13 ± 0.24 Ω^−1^ cm^2^ mol^−1^. These values are well below those reported for electrolytic species and fall within the typical range for non-electrolytes (Λ_m_ < 60 Ω^−1^ cm^2^ mol^−1^ for DMF and Λ_m_ < 50 Ω^−1^ cm^2^ mol^−1^ for DMSO) [58]. Accordingly, complexes **1**–**4** behave as non-electrolytes in both solvents.

The low molar conductivity values indicate that complexes **1**–**4** behave as non-electrolytes under the measurement conditions and are consistent with the absence of substantial dissociation of halides as freely mobile counterions. However, these measurements do not establish the solid-state arrangements observed for the individual complexes, including the monomeric structure of complex 1 and the polymeric and dimeric structures of complexes **3** and **4**, respectively. Dissolution may disrupt bridging M–X interactions and generate oligomeric or discrete species, potentially with solvent molecules coordinated to the metal centers. Therefore, conductivity data support the non-electrolytic behavior of the complexes but do not define the molecular species present in solution.

### 2.3. Spectroscopy

IR spectroscopy was used to investigate the coordination behavior of the thioamide ligands and to confirm the solid-state identity of complexes **1**–**4** (Figures S1–S6). The spectra were recorded in the 4000–400 cm^−1^ region. In the free ligands, the bands at 3089 cm^−1^ (Hmmthz) and 3043 cm^−1^ (Hmmthd) are assigned to N–H stretching vibrations. In the complexes, these bands are observed in the 3230–3087 cm^−1^ region, indicating that the N–H group remains intact and does not undergo deprotonation during coordination.

Additional evidence for coordination is provided by the thioamide-related vibrations. The C–N stretching bands observed at 1446 cm^−1^ (Hmmthd) and 1460 cm^−1^ (Hmmthz) in the free ligands shift to lower wavenumbers (1429–1417 cm^−1^) upon complexation. Similarly, the C–S stretching bands, appearing at 765 cm^−1^ (Hmmthd) and 773 cm^−1^ (Hmmthz) in the free ligands, are shifted to lower frequencies (767–738 cm^−1^) in the complexes. These shifts are consistent with coordination through the sulfur atom of the thione group [59,60].

Raman spectroscopy provides further support for the formation of metal–sulfur and metal–halogen bonds. In the Raman spectra of the antimony(III) complexes (**1** and **2**, Figures S7 and S8), bands in the 355–342 cm^−1^ region are assigned to Sb–S stretching vibrations, while those in the 217–191 cm^−1^ region correspond to Sb–Br modes. For the bismuth(III) complexes (**3** and **4,** Figures S9 and S10), bands in the 358–348 cm^−1^ region are attributed to Bi–S stretching vibrations. Additional bands at 260–241 cm^−1^ (complex **3**) and 188–148 cm^−1^ (complex **4**) are assigned to Bi–Cl and Bi–Br vibrations, respectively. These observations confirm the presence of M–S (M = Sb, Bi) and M–X (X = Cl, Br) bonds.

The ^1^H and ^13^C NMR spectra of complexes **1**–**4**, recorded in DMSO-d_6_, further support these findings (Figures S11–S22). The retention of –NH proton signals at δ ≈ 13.10–14.30 ppm, together with the presence of C=S carbon resonances at δ ≈ 188.49–189.54 ppm, indicates that the ligands remain in their neutral thione form without deprotonation, consistent with the IR results [61,62].

Taken together, the FT-IR, Raman, and NMR spectroscopic results consistently demonstrate that the ligands coordinate to the Sb(III) and Bi(III) centers as neutral monodentate sulfur donors, in full agreement with the crystal structures determined for complexes **1**, **3**, and **4**.

### 2.4. Thermal Analysis

The thermal behavior of the complexes was investigated by TG–DTA analysis under a nitrogen atmosphere in the temperature range of 25–1000 °C (Figures S26–S29). All complexes exhibit thermal stability up to 120–160 °C, followed by decomposition processes associated with the loss of ligand and halide components.

Complexes **1**, **2**, and **4** display similar single-step decomposition patterns. Complex **1** shows a mass loss of 80.77% (calc. 80.48%) in the range 160–380 °C, while complex **2** exhibits a mass loss of 81.41% (calc. 80.61%) between 120 and 225 °C, and complex **4** undergoes decomposition in the range 130–340 °C with a mass loss of 71.12% (calc. 70.70%). In all three cases, the observed mass losses correspond to the simultaneous removal of two ligand molecules and three halide ions. The lower decomposition onset temperature of complex **2** suggests relatively weaker metal–ligand interactions compared to complexes **1** and **4**.

In contrast, complex **3** exhibits a distinct two-step decomposition process. The first step (160–350 °C) corresponds to ligand removal, with a mass loss of 45.87% (calc. 45.42%), while the second step (350–840 °C) is attributed to the elimination of three chloride ions, showing a mass loss of 19.12% (calc. 18.41%). This stepwise behavior indicates different binding strengths for the ligand and halide groups.

At higher temperatures, all complexes leave metal-containing residues that are gradually decomposed upon further heating. The close agreement between experimental and calculated mass losses supports the proposed decomposition pathways. Notably, Sb(III) complexes decompose at relatively lower temperatures, whereas the Bi(III) complexes, particularly complex **3**, exhibit a more stepwise and thermally resistant decomposition profile. This difference can be attributed to the higher polarizability and larger ionic radius of the bismuth center, which likely leads to stronger metal–ligand interactions and highlights the significant role of the metal center in determining thermal stability.

### 2.5. Crystal and Molecular Structures of the Complexes 1, 3, and 4

**Figure 1:**
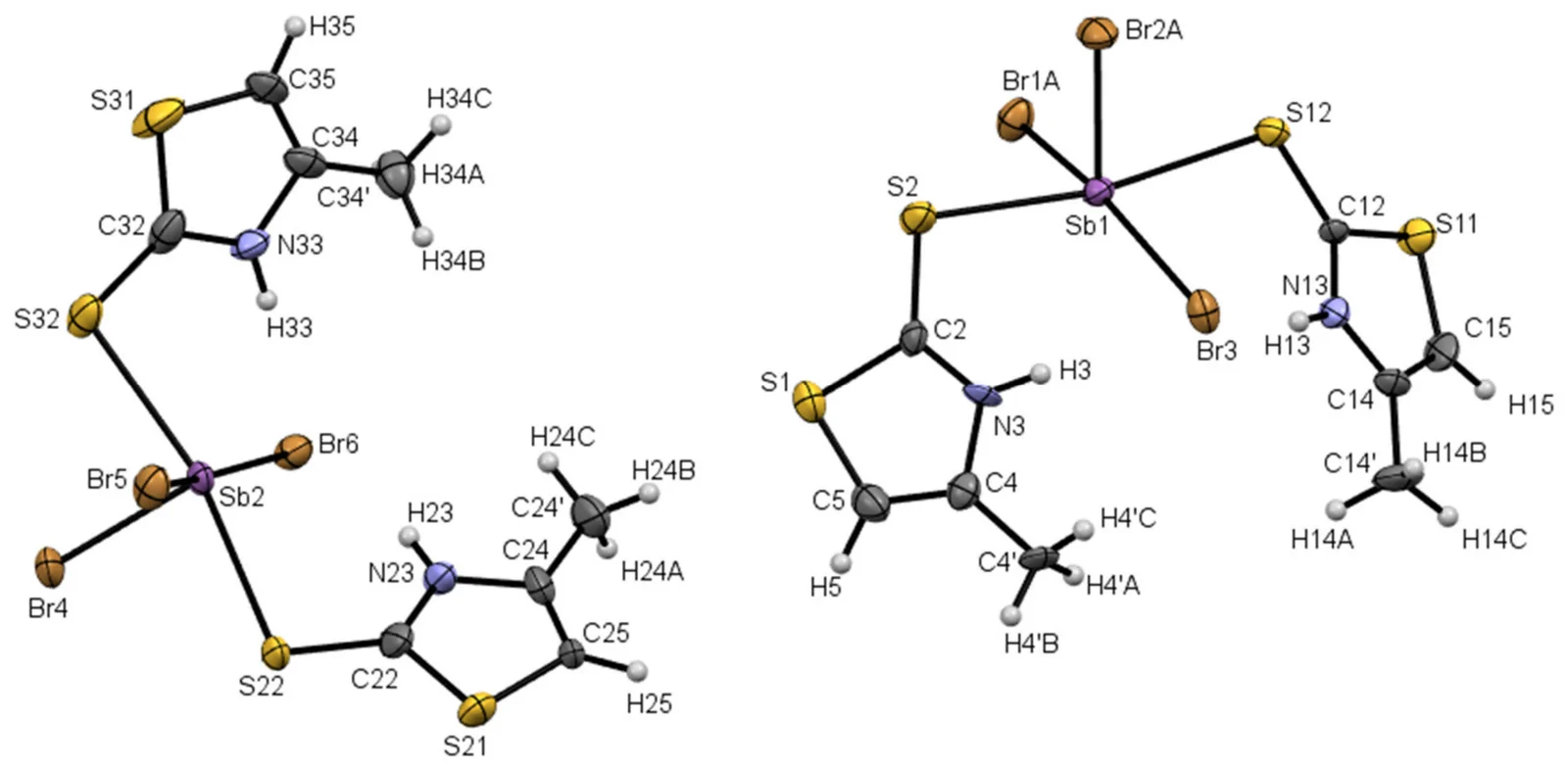
Molecular structure of complex **1**.

**Figure 2:**
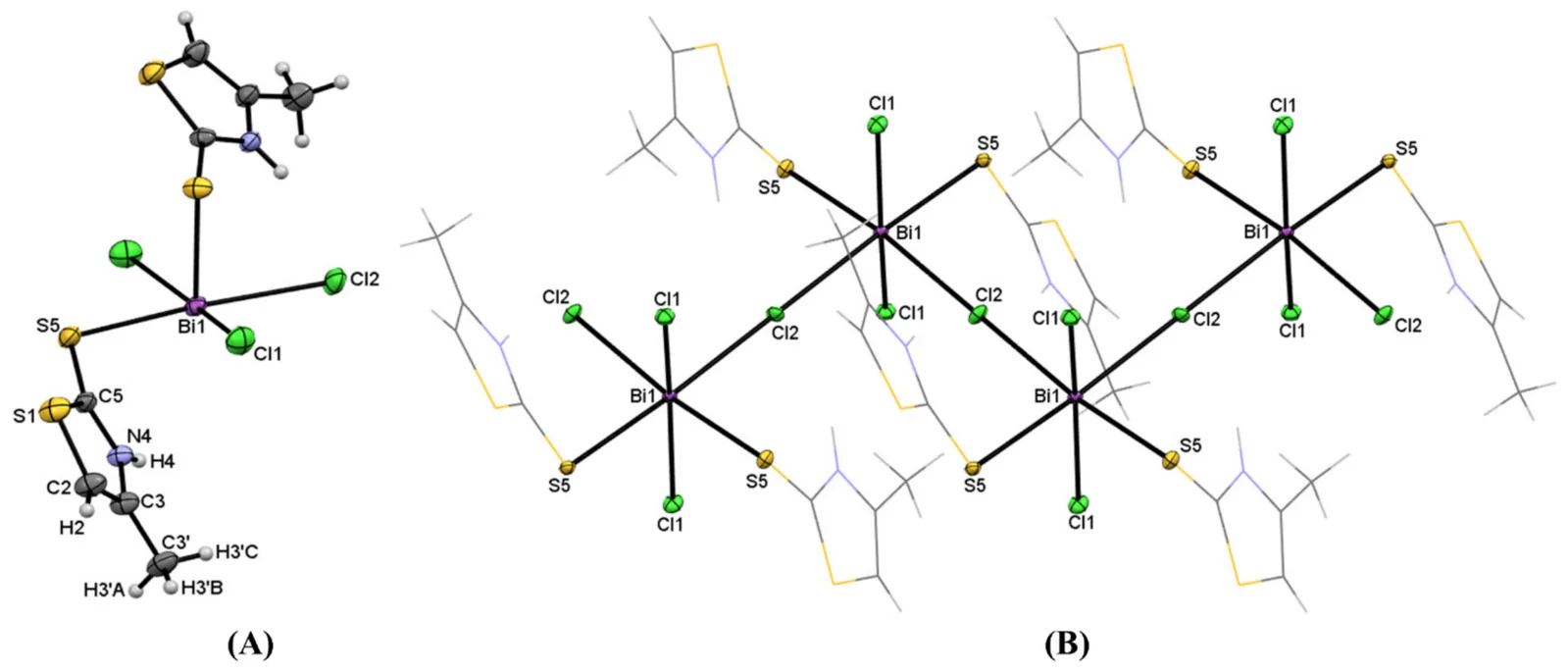
(**A**) Molecular structure of complex **3**. (**B**) Polymeric structure resulting from Bi**^…^**Cl interactions in complex **3**.

**Figure 3:**
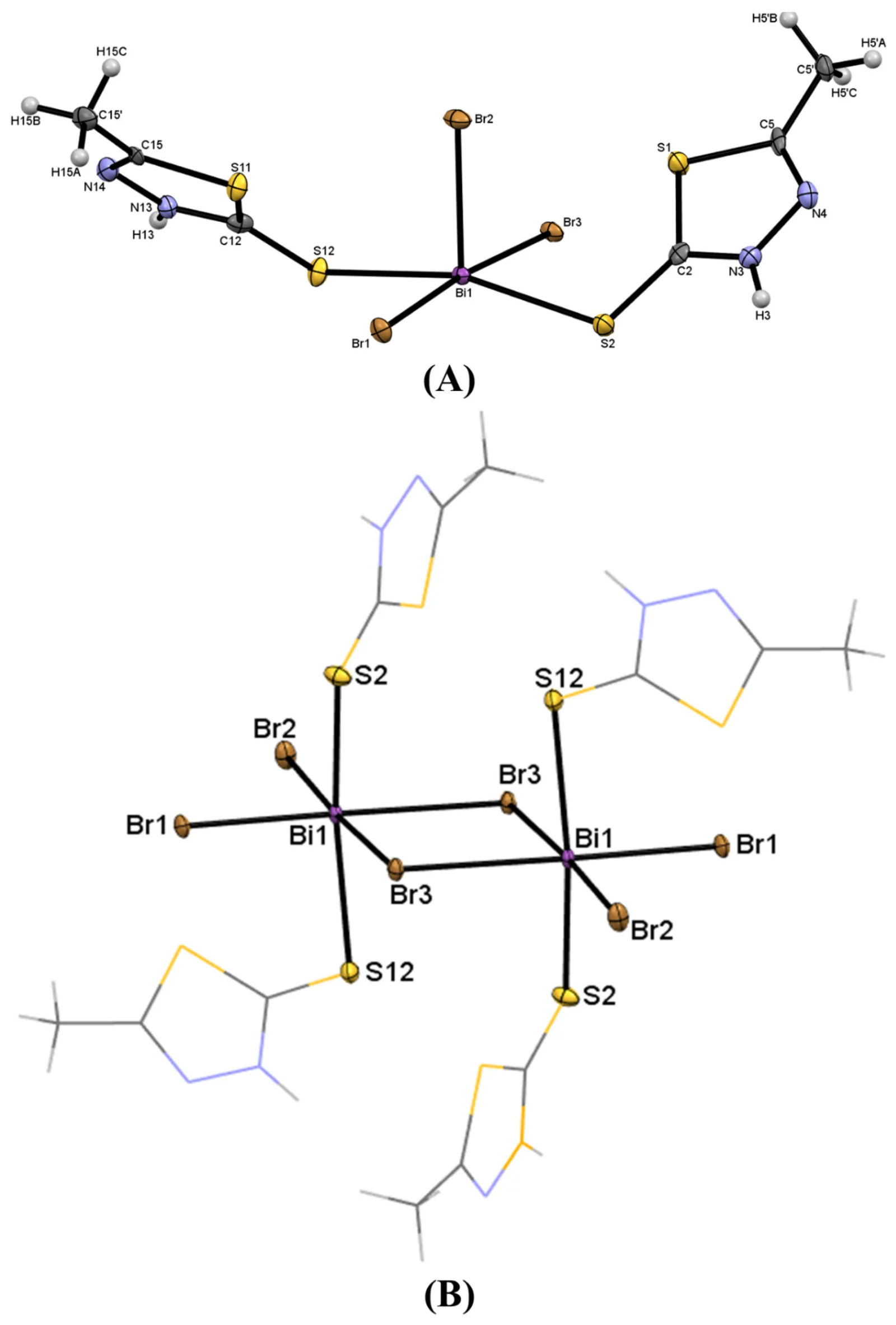
(**A**) Molecular structure of complex **4**. (**B**) Dimeric structure resulting from Bi**^…^**Br interactions in complex **4**.

Single-crystal X-ray diffraction analyses were performed to elucidate the molecular and supramolecular structures of complexes **1**, **3**, and **4**. Crystallographic data collection and refinement details are summarized in Table 1, while the molecular structures with atom-labeling schemes are shown in Figure 1, Figure 2 and Figure 3. Selected bond lengths and angles are listed in Table 1.

To place the present crystal structures into context, the structural features of complexes **1**, **3**, and **4** were compared with previously reported square-pyramidal Sb(III) and Bi(III) halide–thioamide complexes [63,64,65,66,67,68,69]. These compounds commonly adopt three principal coordination arrangements—*cis*-S/*cis*-X, *trans*-S/*trans*-X, and apical-S—depending on the relative positions of the halide and sulfur donor atoms within the coordination sphere. They also exhibit a strong tendency to assemble through bridging interactions, most commonly involving μ_2_-halide ligands, giving rise to dimeric or polymeric architectures. This structural diversity reflects the ability of Sb(III) and Bi(III) centers to expand their coordination environments through secondary bonding interactions, facilitated by the presence of a stereochemically active lone pair. The present structures display distinct solid-state arrangements: complex **1** is monomeric, whereas complexes **3** and **4** form polymeric and dimeric structures, respectively.

The crystal structure of complex **1** consists of discrete [SbBr_3_(Hmmthz)_2_] monomers. Two crystallographically independent monomeric units are present, and both adopt the *trans*-Br/*trans*-S isomeric arrangement. In each unit, the Sb(III) centre is coordinated by two sulfur atoms from two Hmmthz ligands and three bromide ions, giving a distorted square-pyramidal geometry. Br2A and Br4 occupy the apical positions around Sb1 and Sb2, respectively, while the two sulfur atoms and the remaining two bromide ions define the basal planes. The trans angles in the basal plane are S2–Sb1–S12 = 166.7(1)° and Br1A–Sb1–Br3 = 170.72(6)° for Sb1, and S22–Sb2–S32 = 166.3(2)° and Br5–Sb2–Br6 = 170.67(8)° for Sb2. The apical Br–Sb–basal donor angles fall in the range 82.7(1)–96.86(6)°. The Sb–S bond lengths are 2.678(5)–2.838(5) Å. The apical Sb–Br distances are 2.5512(18) and 2.554(2) Å, whereas the basal Sb···Br distances are unequal: 2.5802(19) and 3.212(2) Å for Sb1, and 2.580(2) and 3.220(2) Å for Sb2. The S–C distances of 1.68(2)–1.706(16) Å are consistent with the thione form of the ligands. The bromide ions do not bridge adjacent Sb centres; therefore, the complex retains a monomeric structure in the crystal.

The crystal structure of complex **3** consists of one-dimensional coordination polymeric chains formed by μ_2_-chloride bridges. Each Bi(III) centre is coordinated by two neutral Hmmthz ligands through their thione sulfur atoms, two terminal chloride ions and two bridging chloride donors, resulting in a distorted octahedral coordination environment. The N–H bond is retained, consistent with coordination of the ligands in their neutral thione form. The Bi–S, terminal Bi–Cl and bridging Bi–Cl bond lengths are 2.6724(19), 2.650(2) and 2.9881(4) Å, respectively. The longer Bi–Cl distance associated with the bridging chloride reflects its role in linking adjacent Bi(III) centres. The coordination geometry is distorted, as shown by the Cl1–Bi1–Cl1^1^ and S5–Bi1–Cl2 angles of 163.93(10)° and 174.20(5)°, respectively. The Bi1–Cl2–Bi1^1^ angle is 180°, and the chloride bridges extend the structure into a one-dimensional polymeric chain.

Complex **4**, derived from 2-mercapto-5-methyl-1,3,4-thiadiazole, exhibits a distinct supramolecular organization and adopts the trans-S/trans-Br arrangement. The ligands coordinate in their neutral thione form through the sulfur atoms, with Bi–S bond lengths of 2.821(2) and 2.875(1) Å; the corresponding S–C bond lengths are 1.685(6) and 1.696(6) Å. Neighboring monomeric units are linked through bromide bridges to form centrosymmetric, edge-sharing dimers with distorted octahedral coordination environments. The terminal Bi–Br bond lengths are 2.7374(6) and 2.6584(7) Å, while the Bi–Br distances involving the bridging bromides are 2.9752(6) and 3.0808(7) Å. The trans arrangement of the bromide donors is reflected by the Br1–Bi1–Br3 and Br2–Bi1–Br33 angles of 174.74(2)° and 175.75(2)°, respectively. The Br1–Bi1–Br2 angle is 92.77(2)°, and the Bi1–Br3–Bi13 angle at the bromide bridge is 92.82(2)°. These binuclear units are subsequently interconnected through additional Bi···Br and Bi···S secondary interactions, producing two-dimensional polymeric chains. The resulting framework is further reinforced by weak intermolecular contacts, particularly N–H···Br hydrogen bonds, giving rise to an extended three-dimensional supramolecular network (Table S1).

In the solid state, complex **3** forms a chloride-bridged polymeric structure, whereas complex **4** forms a bromide-bridged dimer. In these complexes, chloride and bromide are coordinated and bridging ligands rather than external counterions. However, complexes **3** and **4** contain different ligands (Hmmthz and Hmmthd, respectively); therefore, their distinct solid-state structures cannot be attributed solely to the identity of the halide.

Overall, the crystal structures of complexes **1**, **3**, and **4** show that metal identity and heterocyclic ligand framework influence the coordination arrangements, secondary bonding interactions, and supramolecular assembly. These solid-state features are compatible with the spectroscopic and molar conductivity results; however, the solution measurements do not establish whether these distinct solid-state arrangements are retained after dissolution. Cleavage of bridging interactions and solvent coordination may lead to solution species that differ from those observed in the solid state.

### 2.6. Stability Studies

The solution stability of the antimony(III) and bismuth(III) halide complexes derived from 2-mercapto-4-methylthiazole (Hmmthz) and 2-mercapto-5-methyl-1,3,4-thiadiazole (Hmmthd) was evaluated using time-dependent molar conductivity and UV–Vis spectroscopy. Conductivity measurements in DMSO at 0, 24, and 48 h remained nearly constant and within the non-electrolyte range, indicating that the complexes preserve their integrity in solution without dissociation of halide ions (Table S2). Consistently, the UV–Vis spectra recorded in both DMSO and phosphate-buffered aqueous medium (pH 7.4) showed no significant shifts in absorption maxima and no emergence of new bands over time (Figures S24 and S25). The absence of significant changes in the UV–Vis spectra and molar conductivity indicates that no substantial changes in these measured properties occurred under the investigated conditions. However, these measurements do not establish whether the solid-state arrangements observed for the individual complexes are retained in solution. Overall, the conductivity and UV–Vis results show no substantial changes in the measured properties under the investigated conditions, including in phosphate-buffered aqueous medium at pH 7.4.

### 2.7. Antiproliferative Study

The antiproliferative activities of the ligands (Hmmthz and Hmmthd) and their corresponding Sb(III) and Bi(III) complexes (**1**–**4**) were evaluated against the human cervical cancer cell line (HeLa) using the Sulforhodamine B (SRB) assay. The IC_50_ values are summarized in Table 2 together with that of cisplatin, which was evaluated as a positive control under the same experimental conditions, while the literature-reported value for carboplatin is included for contextual comparison [70]. The corresponding dose–response curves are provided in the Supplementary Materials (Figure S30).

The results indicate that the Sb(III) complexes **1** and **2** exhibited antiproliferative activity against HeLa cells, with IC_50_ values of 8.5 ± 0.3 and 7.9 ± 0.3 μM, respectively. Under the same experimental conditions, cisplatin exhibited an IC_50_ value of 3.9 ± 0.1 μM, indicating higher antiproliferative activity than complexes **1** and **2**. In contrast, the Bi(III) complexes **3** and **4** and the free ligands Hmmthz and Hmmthd were inactive within the investigated concentration range (IC_50_ > 30 μM). Within the limited series investigated, these results reveal differences in antiproliferative activity between the Sb(III) and Bi(III) complexes; however, the contribution of the metal center cannot be considered independently of other structural differences among the compounds.

The biological activity of the pnictogen starting materials should also be considered in this context. SbBr_3_ has previously been reported to exhibit antiproliferative activity against HeLa cells, with an IC_50_ value of 12.1 ± 0.7 μM [64]. Complexes **1** and **2** showed lower IC_50_ values than the reported value for SbBr_3_, while the corresponding free ligands were inactive within the investigated concentration range (IC_50_ > 30 μM). Within the limitations of this comparison, these observations suggest that complex formation may modify the antiproliferative response of the Sb(III) system. However, the present data do not allow the relative contributions of the Sb(III) center and the coordinated ligand environment to be independently established.

Differences in antiproliferative activity between Sb(III)- and Bi(III)-containing sulfur-donor complexes have also been observed in related systems [64,68,71]. Although differences in antiproliferative activity are observed between the Sb(III) and Bi(III) complexes in the present series, the limited dataset does not allow the individual contributions of metal identity, ligand structure, halide composition, and supramolecular organization to be clearly distinguished.

In addition to differences in metal and ligand composition, the crystallographically characterized complexes exhibit distinct solid-state arrangements. Complex **1** is monomeric, whereas complex **3** forms a chloride-bridged polymer and complex **4** forms bromide-bridged dimeric units. By comparison, previously reported ionic Sb(III) complexes, such as {[SbCl_2_(Hmtt)_4_]·Cl} and {[SbBr_2_(Hmtt)_4_]·Br}, are monomeric and exhibit IC_50_ values of 9.1 ± 0.5 and 15.7 ± 0.5 μM, respectively [64]. The differences in antiproliferative activity observed among these structurally distinct compounds indicate that structural organization may be considered as one of several potentially relevant factors; however, the available dataset is insufficient to establish a direct relationship between supramolecular architecture and antiproliferative activity.

Overall, the present results reveal differences in antiproliferative activity among the investigated compounds; however, the limited number of complexes does not allow the individual effects of metal identity, ligand structure, halide composition, and supramolecular organization to be clearly separated. Further studies involving additional cancer cell lines as well as appropriate non-malignant human cells will be required to determine whether the antiproliferative activity observed for complexes **1** and **2** extends beyond HeLa cells and whether these compounds exhibit selectivity toward cancer cells. In the absence of data from non-malignant cells, no conclusions regarding cancer-cell selectivity or selectivity indices can be drawn from the present study.

### 2.8. Antimicrobial Study

**Figure 4:**
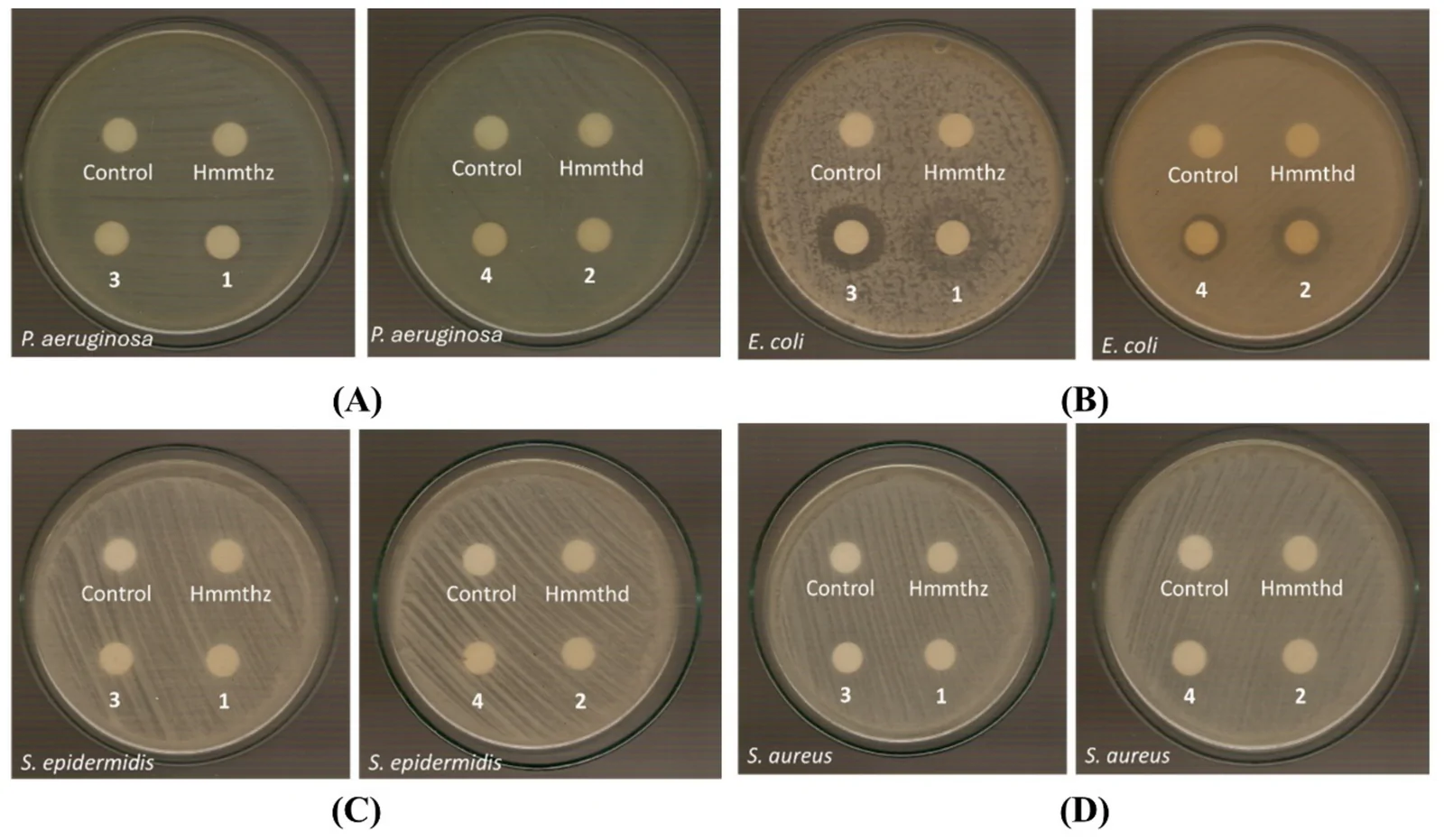
Representative agar plates showing the inhibition zones for (**A**) *P. aeruginosa*, (**B**) *E. coli*, (**C**) *S. epidermidis*, and (**D**) *S. aureus* following treatment with complexes **1**–**4** and their corresponding free ligands at 1 mM.

The antibacterial effects of complexes **1**–**4** and the corresponding free ligands were evaluated as a preliminary screening assay against the Gram-negative bacteria *P. aeruginosa* and *E. coli* and the Gram-positive bacteria *S. epidermidis* and *S. aureus* using the agar disc-diffusion method at a concentration of 1 mM. Ciprofloxacin was included as a positive control under the same experimental conditions. The resulting inhibition-zone (IZ) values, together with those of structurally related compounds reported in the literature, are summarized in Table 3, while representative agar plates for complexes **1**–**4** and the corresponding free ligands are presented in Figure 4. Discs (9 mm in diameter) loaded with the tested compounds were placed on inoculated agar plates and incubated at 37 °C for 20 h.

The free ligands Hmmthz and Hmmthd showed no measurable inhibition beyond the disc diameter (9.0 mm) against any of the tested bacterial strains. Among complexes **1**–**4**, the most pronounced inhibition was observed against *E. coli*, with IZ values of 10.5 ± 0.9, 12.8 ± 0.5, 16.0 ± 1.1, and 12.5 ± 0.9 mm for complexes **1**–**4**, respectively. Complexes **1** and **2** produced only minor inhibition against *P. aeruginosa* (9.3 ± 0.5 mm), whereas complexes **3** and **4** showed no inhibition beyond the disc diameter against this strain. No inhibition beyond the disc diameter was observed against *S. epidermidis* for any of the four complexes, while only complex 4 produced a minor inhibition-zone response against *S. aureus* (9.5 ± 0.9 mm).

The difference in the *E. coli* inhibition zones between the Bi(III) complexes **3** and **4** should be interpreted cautiously. Although complex **3** contains chloride and complex **4** contains bromide, the two complexes also differ in ligand identity (Hmmthz and Hmmthd, respectively) and solid-state structure. Therefore, the observed difference cannot be assigned solely to the halide, and the present results do not establish a halide-dependent antibacterial trend. A systematic assessment of the halide effect would require a matched series containing the same ligand and comparable coordination structures.

Under the same experimental conditions, ciprofloxacin produced IZ values of 35.5 ± 0.6, 33.0 ± 0.8, 39.2 ± 0.9, and 30.5 ± 0.6 mm against *P. aeruginosa*, *E. coli*, *S. epidermidis*, and *S. aureus*, respectively. Thus, the inhibition zones produced by complexes **1**–**4** were considerably smaller than those observed for ciprofloxacin. Nevertheless, coordination of Hmmthz and Hmmthd was associated with increased inhibition-zone responses against *E. coli* relative to the corresponding free ligands, with complex **3** showing the largest inhibition zone (16.0 ± 1.1 mm) among the investigated complexes.

Several previously reported Sb(III) and Bi(III) sulfur-donor complexes have also shown their most pronounced inhibition-zone responses against Gram-negative bacteria, particularly *E. coli*. For example, the Hmtt-containing Sb(III) complexes [SbCl_3_(Hmtt)_2_] and [SbBr_3_(Hmtt)_2_] exhibited IZ values of 20.0 and 20.5 mm, respectively, against *E. coli* [64]. The related Sb(III) complexes [SbCl_2_(Hatetsc)] and [SbBr_2_(Htmtsc)_2_] produced IZ values of 16.2 and 17.4 mm, respectively [72], whereas the Bi(III) complexes [BiCl_3_(Hattsc)_3_] and [BiBr_3_(Hattsc)_2_] showed more modest responses of 12.5 and 12.0 mm, respectively [73]. These literature results show that antibacterial responses vary among structurally related Sb(III) and Bi(III) complexes and do not, by themselves, establish a general effect of metal identity or halide composition.

**Table 3:** Inhibition-zone (IZ) values (mm) of complexes **1**–**4**, their corresponding free ligands, related compounds, and ciprofloxacin as a positive control against *P. aeruginosa*, *E. coli*, *S. epidermidis*, and *S. aureus*. Values obtained in the present study are expressed as mean ± SD from three independent experiments (*n* = 3).

Compounds	*P. aeruginosa*	*E. coli*	*S. epidermidis*	*S. aureus*	
Hmmthd	9.0 ± 0.0	9.0 ± 0.0	9.0 ± 0.0	9.0 ± 0.0	*
Hmmthz	9.0 ± 0.0	9.0 ± 0.0	9.0 ± 0.0	9.0 ± 0.0	*
**1**	9.3 ± 0.5	10.5 ± 0.9	9.0 ± 0.0	9.0 ± 0.0	*
**2**	9.3 ± 0.5	12.8 ± 0.5	9.0 ± 0.0	9.0 ± 0.0	*
**3**	9.0 ± 0.0	16.0 ± 1.1	9.0 ± 0.0	9.0 ± 0.0	*
**4**	9.0 ± 0.0	12.5 ± 0.9	9.0 ± 0.0	9.5 ± 0.9	*
Hmtt	9.0 ± 0.0	9.0 ± 0.0	9.0 ± 0.0	9.0 ± 0.0	[64]
[SbCl_3_(Hmtt)_2_]	9.9 ± 0.8	20.0 ± 0.0	9.0 ± 0.0	9.3 ± 0.5	[64]
[SbBr_3_(Hmtt)_2_]	10.7 ± 0.7	20.5 ± 2.0	9.0 ± 0.0	9.3 ± 0.5	[64]
[SbI_3_(Hmtt)_2_]	9.0 ± 0.0	10.1 ± 0.6	9.0 ± 0.0	9.0 ± 0.0	[64]
{[SbCl_2_(Hmtt)_4_]⋅Cl}	11.0 ± 0.0	19.0 ± 1.1	9.0 ± 0.0	9.3 ± 0.5	[64]
{[SbBr_2_(Hmtt)_4_]⋅Br}	9.0 ± 0.0	9.7 ± 0.7	9.0 ± 0.0	9.0 ± 0.0	[64]
[BiCl_3_(Hattsc)_3_]	12.0 ± 0.6	12.5 ± 0.6	9.0 ± 0.0	9.0 ± 0.0	[73]
[BiBr_3_(Hattsc)_2_]	13.2 ± 0.9	12.0 ± 0.8	9.0 ± 0.0	9.0 ± 0.0	[73]
[SbCl_2_(Hatetsc)]	14.8 ± 1.1	16.2 ± 0.7	9.0 ± 0.0	9.0 ± 0.0	[72]
[SbBr_2_(Htmtsc)_2_]	11.2 ± 1.2	17.4 ± 1.7	9.8 ± 0.9	9.0 ± 0.0	[72]
Ciprofloxacin	35.5 ± 0.6	33 ± 0.8	39.2 ± 0.9	30.5 ± 0.6	*

* In this study, *P. aeruginosa* = *Pseudomonas aeruginosa*, *E. coli* = *Escherichia coli*, *S. epidermidis* = *Staphylococcus epidermidis*, *S. aureus* = *Staphylococcus aureus*.

Overall, the present results should be regarded as preliminary inhibition-zone-based antibacterial screening data. Although complex formation was associated with increased inhibition-zone responses against *E. coli* relative to the free ligands, the considerably smaller inhibition zones compared with ciprofloxacin and the absence or very limited responses against the other tested strains do not support conclusions regarding potent or selective antibacterial activity. Quantitative evaluation by standardized MIC assays would be required to establish the antibacterial potency of these compounds.

## 3. Experimental

### 3.1. Materials and Instruments

Antimony(III) and bismuth(III) halides were purchased from Sigma-Aldrich (St. Louis, MO, USA), while 2-mercapto-4-methylthiazole (Hmmthz) and 2-mercapto-5-methyl-1,3,4-thiadiazole (Hmmthd) were obtained from Thermo Fisher Scientific (Waltham, MA, USA). All solvents were purchased from Merck (Darmstadt, Germany). All reagents and solvents were used as received without further purification. Melting points were determined using a Stuart SMP30 digital melting point apparatus (Bibby Scientific Ltd., Stone, UK). Electrical conductivity measurements were carried out at ambient temperature using a VWR Phenomenal CO 3000 L conductivity meter (VWR International, Leuven, Belgium). Elemental analyses (C, H, N, and S) were performed on a Carlo Erba EA 1108 analyzer (Carlo Erba Instruments, Milan, Italy). Infrared spectra were recorded in the range 4000–400 cm^−1^ using a Bruker Vertex 70 FT-IR spectrometer equipped with an ATR module (Bruker Optik GmbH, Ettlingen, Germany). Raman spectra were obtained on a Renishaw InVia Raman microscope (Renishaw plc, Wotton-under-Edge, UK). UV–Vis absorption spectra were recorded using a Shimadzu UV-2600 spectrophotometer (Shimadzu Corporation, Kyoto, Japan). ^1^H and ^13^C NMR spectra were measured in DMSO-*d*_6_ on a Varian Unity Inova 400 MHz spectrometer (Varian, Inc., Palo Alto, CA, USA), using tetramethylsilane (TMS) as an internal reference, with chemical shifts reported in ppm. Thermal analyses (TG–DTA) were carried out on a Seiko SII TG/DTA 7200 system (SII NanoTechnology Inc., Chiba, Japan) under a nitrogen flow (50 cm^3^ min^−1^) at a heating rate of 5 °C min^−1^.

### 3.2. Single Crystal X-Ray Crystallography

Single-crystal X-ray diffraction data (Table 4) for complexes **1**, **3**, and **4** were collected using different diffractometers. Data for **1** were collected on a Rigaku SuperNova diffractometer (Rigaku Oxford Diffraction, Yarnton, UK) equipped with an Atlas CCD detector using mirror-monochromated CuKα radiation (λ = 1.54184 Å) at 130 K. Data for **3** were collected on a Bruker D8 QUEST diffractometer (Bruker AXS Inc., Madison, WI, USA) equipped with a microfocus sealed tube (MoKα radiation, λ = 0.71073 Å), a multilayer mirror monochromator, and a Bruker PHOTON III CPAD detector at 273 K. Data for **4** were collected on a Rigaku Xcalibur four-circle diffractometer equipped with an Eos detector using graphite-monochromated MoKα radiation (λ = 0.71073 Å) at 100 K. For complexes **1** and **4**, data were collected using the ω-scan technique [74]. Lorentz–polarization and absorption corrections were applied to the diffraction data [75]. For complex **4**, the intensity data were integrated using SAINT V8.41 [76], and a multi-scan absorption correction was applied using SADABS 2016/2 [77]. Unit-cell parameters were refined by least-squares fitting of the measured reflections. The crystal structures were solved using SHELXT [78] and refined by full-matrix least-squares methods on *F*2 using SHELXL-2015/2017 [79] within the OLEX2 [80] and WinGX [81] program packages. All non-hydrogen atoms were refined anisotropically. Hydrogen atoms were placed in calculated positions and refined using a riding model with isotropic displacement parameters fixed at 1.2*U*eq of their parent atoms. Complex **1** was refined as a non-merohedral twin using an HKLF 5 file. The refined BASF parameter converged to 0.5199(19), corresponding to an approximately 52:48 twin domain ratio. In addition, four atoms were modelled as disordered over two positions with refined site-occupancy factors of approximately 0.90/0.10, and both disorder components were refined anisotropically. In complex **3**, disorder was identified and partially modelled. The structural model was refined with all heavy atoms fully modelled. The organic ligand exhibits unresolved disorder; however, owing to the limited quality of the diffraction data, the alternative ligand position, estimated to have an occupancy of approximately 15%, could not be reliably identified and modelled. This minor component is likely to overlap partially with the major ligand position. Crystallographic data for complexes **1**, **3**, and **4** have been deposited with the Cambridge Crystallographic Data Centre (CCDC) under deposition numbers 2,534,028 (**1**), 2,534,029 (**3**), and 2,534,027 (**4**).

### 3.3. General Procedure for the Synthesis of Complexes **1**–**4**

2-Mercapto-4-methylthiazole (Hmmthz: 0.131 g, 1.0 mmol) or 2-mercapto-5-methyl-1,3,4-thiadiazole (Hmmthd: 0.132 g, 1.0 mmol) was dissolved in methanol for complex **1** or dichloromethane for complexes **2**–**4** (10 mL). Antimony(III) bromide (0.181 g, 0.5 mmol) and bismuth(III) chloride or bismuth(III) bromide (0.158–0.224 g, 0.5 mmol) were dissolved in acetonitrile for complex **1** or dichloromethane for complexes **2**–**4** (10 mL). The ligand solutions were added dropwise to the metal salt solutions under continuous stirring. Clear solutions were obtained for complexes **1**–**3** (light yellow, yellow, and orange, respectively), whereas complex **4** formed a yellow precipitate. All reaction mixtures were stirred at room temperature for 3 h. Although the solutions for complexes **1**–**3** appeared clear, they were passed through filter paper as a precaution to remove any trace undissolved material or fine suspended particles before being allowed to evaporate slowly in the dark at ambient temperature. Yellow or orange crystals were obtained after approximately two weeks. For complex **4**, the precipitate was filtered, dried under vacuum, redissolved in hot acetonitrile, and then allowed to crystallize in the dark, affording yellow crystals within two weeks.

[SbBr_3_(Hmmthz)_2_] (**1**): yellow crystals, yield 83% (0.258 g), m.p.: 165–168 °C, elemental analysis calculated (found) for C_8_H_10_Br_3_N_2_S_4_Sb; C: 15.40 (15.33), H: 1.62 (1.59), N: 4.49 (4.43), S: 20.55 (20.62). Λ_M_ (DMSO): 11.25 ± 0.08 Ω^−1^cm^2^mol^−1^. Λ_M_ (DMF): 6.56 ± 0.16 Ω^−1^cm^2^mol^−1^. FT-IR (cm^−1^): 3087w ν(N-H), 1417s ν(C=N), 763m ν(C=S). FT-Raman (cm^−1^): 355 ν(Sb-S), 217, 200 ν(Sb-Br). ^1^H NMR (400 MHz, DMSO-d_6_, δ, ppm): 13.14 (s, 2H, -NH), 6.57 (s, 2H, =C-H), 2.14 (s, 6H, -CH_3_). ^13^C NMR (100 MHz, DMSO-d_6_, δ, ppm): 189.36 (C=S), 139.80 (C-N), 109.37 (C-S), 14.43 (-CH_3_). UV-Vis (DMSO, nm) λ_max_ (logε): 253 (4.12), 323 (4.53), (Figure S23).

[SbBr_3_(Hmmthd)_2_] (**2**): yellow crystals, yield 79% (0.247 g), m.p.: 120–122 °C, elemental analysis calculated (found) for C_6_H_8_Br_3_N_4_S_4_Sb; C: 11.51 (11.43), H: 1.29 (1.27), N: 8.95 (8.90), S: 20.49 (20.55). Λ_M_ (DMSO): 12.01 ± 0.21 Ω^−1^cm^2^mol^−1^. Λ_M_ (DMF): 6.93 ± 0.37 Ω^−1^cm^2^mol^−1^. FT-IR (cm^−1^): 3228w ν(N-H), 1429s ν(C=N), 739m ν(C=S). FT-Raman (cm^−1^): 342 ν(Sb-S), 210, 191 ν(Sb-Br). ^1^H NMR (400 MHz, DMSO-d_6_, δ, ppm): 14.30 (s, 2H, -NH), 2.48 (s, 6H, -CH_3_). ^13^C NMR (100 MHz, DMSO-d_6_, δ, ppm): 189.54 (C=S), 160.59 (C=N), 17.12 (-CH_3_). UV-Vis (DMSO, nm) λ_max_ (logε): 253 (4.05), 310 (4.54), (Figure S23).

[{BiCl_2_(µ_2_-Cl)(Hmmthz)_2_}_n_] (**3**): orange crystals, yield 86% (0.248 g), m.p.: 154–156 °C, elemental analysis calculated (found) for C_8_H_10_Cl_3_N_2_S_4_Bi; C: 16.63 (16.55), H: 1.74 (1.71), N: 4.85 (4.80), S: 22.20 (22.27). Λ_M_ (DMSO): 12.68 ± 0.35 Ω^−1^cm^2^mol^−1^. Λ_M_ (DMF): 5.42 ± 0.21 Ω^−1^cm^2^mol^−1^. FT-IR (cm^−1^): 3186w ν(N-H), 1417s ν(C=N), 767m ν(C=S). FT-Raman (cm^−1^): 358 ν(Bi-S), 260, 241 ν(Bi-Cl). ^1^H NMR (400 MHz, DMSO-d_6_, δ, ppm): 13.10 (s, 2H, -NH), 6.53 (s, 2H, =C-H), 2.11 (s, 6H, -CH_3_). ^13^C NMR (100 MHz, DMSO-d_6_, δ, ppm): 189.03 (C=S), 139.96 (C-N), 107.83 (C-S), 15.69 (-CH_3_). UV-Vis (DMSO, nm) λ_max_ (logε): 253 (4.30), 322 (4.60), (Figure S23).

[{BiBr_2_(µ_2_-Br)(Hmmthd)_2_}_2_] (**4**): yellow crystals, yield 78% (0.278 g), m.p.: 133–135 °C, elemental analysis calculated (found) for C_6_H_8_Br_3_N_4_S_4_Bi; C: 10.11 (10.05), H: 1.13 (1.11), N: 7.86 (7.81), S: 17.98 (18.04). Λ_M_ (DMSO): 19.13 ± 0.24 Ω^−1^cm^2^mol^−1^. Λ_M_ (DMF): 10.12 ± 0.74 Ω^−1^cm^2^mol^−1^. FT-IR (cm^−1^): 3230w ν(N-H), 1421s ν(C=N), 746m ν(C=S). FT-Raman (cm^−1^): 348 ν(Bi-S), 188, 148 ν(Bi-Br). ^1^H NMR (400 MHz, DMSO-d_6_, δ, ppm): 14.30 (s, 2H, -NH), 2.48 (s, 6H, -CH_3_). ^13^C NMR (100 MHz, DMSO-d_6_, δ, ppm): 188.49 (C=S), 160.85 (C=N), 18.01 (-CH_3_). UV-Vis (DMSO, nm) λ_max_ (logε): 254 (4.38), 310 (4.55), (Figure S23).

### 3.4. Biological Studies

#### 3.4.1. Antiproliferative Studies

The antiproliferative activities of complexes **1**–**4** and the corresponding free ligands (Hmmthz and Hmmthd) against human cervical cancer (HeLa) cells were evaluated using the Sulforhodamine B (SRB) assay, following previously reported procedures [64]. HeLa cells were kindly provided by Dr Georgios Geromichalos (Cell Culture, Molecular Modeling and Drug Design Laboratory, Symeonidion Research Center, Theagenion Cancer Hospital, Thessaloniki, Greece). Stock solutions of complexes **1**–**4** and the free ligands (0.01 M) were freshly prepared in DMSO and subsequently diluted with Dulbecco’s Modified Eagle’s Medium (DMEM) to the desired concentrations (0.5–30 μM). The final concentration of DMSO in the cell culture medium was maintained within the range of 0.02–0.2% (*v*/*v*). The cells were exposed to the tested compounds for 48 h, after which cell viability was determined using the SRB assay. The antiproliferative activity was expressed in terms of IC_50_ values, defined as the concentration of the tested compound required to inhibit cell growth by 50% relative to the untreated control. Cisplatin was evaluated as an experimental positive control under the same assay conditions.

#### 3.4.2. Determination of the Inhibition Zone (Iz) Through Agar Disc-Diffusion Method

The antibacterial activities of complexes **1**–**4** and the corresponding free ligands (Hmmthz and Hmmthd) were evaluated against the Gram-negative bacterial strains *Pseudomonas aeruginosa* (*P. aeruginosa*) and *Escherichia coli* (*E. coli*) and the Gram-positive bacterial strains *Staphylococcus epidermidis* (*S. epidermidis*) and *Staphylococcus aureus* (*S. aureus*) using the agar disc-diffusion method, following previously reported procedures [82]. The bacterial strains were plated onto trypticase soy agar and incubated at 37 °C for 18–24 h. Three to five isolated colonies with similar morphological appearance were selected from a fresh agar plate using a sterile loop and transferred into a tube containing 2 mL of sterile saline solution. The optical density at 620 nm was adjusted to 0.1, corresponding to approximately 10^8^ CFU/mL. For determination of the inhibition zones (IZ), filter-paper discs approximately 9 mm in diameter were loaded with 1 mM solutions of complexes **1**–**4** and the corresponding free ligands and placed on the inoculated agar surface. The plates were subsequently incubated at 37 °C for 20 h, after which the inhibition-zone diameters were measured in millimeters. Ciprofloxacin was included as an experimental positive control.

#### 3.4.3. Statistical Analysis

All quantitative biological experiments were performed in three independent experiments (*n* = 3). The results are expressed as mean ± standard deviation (SD).

## 4. Conclusions

Four new antimony(III) and bismuth(III) halide complexes containing the sulfur-donor ligands Hmmthz and Hmmthd were synthesized and comprehensively characterized by elemental analysis, FT-IR, Raman, UV–Vis, NMR spectroscopy, thermal analysis, molar conductivity measurements, and, for complexes **1**, **3**, and **4**, single-crystal X-ray diffraction. The combined spectroscopic and crystallographic results confirmed that the ligands coordinate to the metal centers as neutral monodentate sulfur donors in their thione form. Structural analyses revealed distinct coordination and supramolecular arrangements depending on the metal–ligand–halide combination, including monomeric, dimeric and polymeric architectures. Conductivity and UV–Vis measurements showed no substantial changes in the measured properties under the investigated conditions, including phosphate-buffered aqueous medium at pH 7.4; however, these results do not establish that the solid-state arrangements, including the monomeric structure of complex 1 and the polymeric and dimeric structures of complexes 3 and 4, respectively, are retained in solution. Biological evaluation showed that the Sb(III) complexes **1** and **2** exhibited antiproliferative activity against HeLa cells, with IC_50_ values of 8.5 ± 0.3 and 7.9 ± 0.3 μM, respectively, whereas the corresponding free ligands Hmmthz and Hmmthd were inactive within the investigated concentration range (IC_50_ > 30 μM). In contrast, the Bi(III) complexes **3** and **4**, like the corresponding free ligands, were inactive within the same concentration range. Thus, within the present series, coordination to Sb(III) was associated with an increase in antiproliferative activity relative to the free ligands, whereas a comparable effect was not observed upon coordination to Bi(III). However, because metal identity, ligand structure, halide composition, and supramolecular organization vary among the investigated complexes, the individual contribution of the metal center cannot be isolated from the other structural factors. The complexes exhibited modest inhibition-zone responses in the antibacterial screening assay, with the most pronounced effects observed against *E. coli*. However, the inhibition zones produced by complexes **1**–**4** were considerably smaller than those observed for ciprofloxacin under the same experimental conditions, while only minor or no inhibition was observed against the other tested bacterial strains. Accordingly, these findings should be regarded as preliminary inhibition-zone-based antibacterial screening results rather than evidence of potent or selective antibacterial activity. Since non-malignant human cells were not included in the present study, the selectivity of the observed antiproliferative activity toward cancer cells cannot be assessed. Further studies involving additional cancer cell lines and appropriate non-malignant human cells will therefore be required to determine whether the antiproliferative activity observed for complexes **1** and **2** extends beyond HeLa cells and whether these compounds exhibit selectivity toward cancer cells. Similarly, standardized MIC determinations would be required for a quantitative assessment of the antibacterial potency of the investigated complexes. Overall, the present study demonstrates the structural diversity of sulfur-donor Sb(III) and Bi(III) halide complexes and provides preliminary information on their antiproliferative and antibacterial properties. The observed differences among the investigated compounds provide a basis for further systematic studies aimed at clarifying how metal identity, ligand structure, halide composition, and structural organization contribute to their biological responses.

## Figures and Tables

**Table 1:** Selected bond lengths (Å) and angles (◦) for the complexes **1**, **3**, and **4**.

1				3		4	
Bond lengths (Å)				Bond lengths (Å)		Bond lengths (Å)	
Sb1-Br1A	2.5802(19)	Sb2-S22	2.678(5)	Bi1-Cl1	2.650(2)	Bi1-Br1	2.7374(6)
Sb1-Br2A	2.5512(18)	Sb2-S32	2.838(5)	Bi1-Cl2	2.9881(4)	Bi1-Br2	2.6584(7)
Sb1-Br3	3.212(2)	S2-C2	1.706(15)	Bi1-S5	2.6724(19)	Bi1-Br3	2.9752(6)
Sb2-Br4	2.554 (2)	S12-C12	1.686(13)	S5-C5	1.727(7)	Bi1-S2	2.821(2)
Sb2-Br5	2.580 (2)	S22-C22	1.706(16)			Bi1-S12	2.875(1)
Sb2-Br6	3.220(2)	S32-C32	1.68(2)			S2-C2	1.685(6)
Sb1-S2	2.838(4)					S12-C12	1.696(6)
Sb1-S12	2.691(4)					Bi1-Br3 ^3^	3.0808(7)
Bond angles (°)				Bond angles (°)		Bond angles (°)	
Br1A-Sb1-Br2A	92.40(7)	Br4-Sb2-Br5	91.41(8)	Cl1-Bi1-Cl2	95.64(5)	Br1-Bi1-Br2	92.77(2)
Br1A-Sb1-Br3	170.72(6)	Br4-Sb2-Br6	91.41(8)	Cl1-Bi1-S5	87.54(6)	Br1-Bi1-Br3	174.74(2)
Br1A-Sb1-S2	90.5(1)	Br4-Sb2-S22	82.9(1)	Cl1-Bi1-Cl1 ^1^	163.93(10)	Br1-Bi1-S2	83.78(3)
Br1A-Sb1-S12	92.7(1)	Br4-Sb2-S32	83.7(1)	Cl1-Bi1-Cl2 ^1^	96.18(5)	Br1-Bi1-S12	95.86(3)
Br2A-Sb1-Br3	96.86(6)	Br5-Sb2-Br6	170.67(8)	Cl1-Bi1-S51	81.67(6)	Br2-Bi1-Br3	92.36(2)
Br2A-Sb1-S2	84.3(1)	Br5-Sb2-S22	92.2(1)	Cl2-Bi1-Cl1 ^1^	96.18(5)	Br2-Bi1-S2	97.88(3)
Br2A-Sb1-S12	82.7(1)	Br5-Sb2-S32	91.2(1)	Cl2-Bi1-Cl2 ^1^	85.148(15)	Br2-Bi1-S12	98.28(3)
Br3-Sb1-S2	91.2(1)	Br6-Sb2-S22	88.0(1)	Cl2-Bi1-S5 ^1^	89.69(4)	Br3-Bi1-S2	94.35(3)
Br3-Sb1-S12	87.8(1)	Br6-Sb2-S32	90.7(1)	S5-Bi1-Cl1 ^1^	81.67(6)	Br3-Bi1-S12	84.56(3)
S2-Sb1-S12	166.7(1)	S22-Sb2-S32	166.3(2)	S5-Bi1-Cl2 ^1^	89.69(4)	Br1-Bi1-Br3 ^3^	87.62(2)
				S5-Bi1-S5 ^1^	95.59(9)	Br2-Bi1-Br3 ^3^	175.75(2)
				Bi1-Cl2-Bi1 ^2^	180.00	Br3-Bi1-Br3 ^3^	87.18(2)
						S2-Bi1-Br3 ^3^	77.94(3)
						S12-Bi1-Br3 ^3^	85.89(3)
						Bi1-Br3-Bi1 ^3^	92.82(2)

^1^ −x + 1, y, −z + 1/2; ^2^ −x + 1, −y + 1, −z; ^3^ 1−x, −y, 1−z.

**Table 2:** The IC_50_ values (μM) of the antimony(III) and bismuth(III) complexes (**1**–**4**) and their free ligands (Hmmthz and Hmmthd) against human cervical cancer (HeLa) cells. Values obtained in the present study are expressed as mean ± SD from three independent experiments (*n* = 3).

Compounds	IC_50_ (μM)	Ref.
**1**	8.5 ± 0.3	*
**2**	7.9 ± 0.3	*
**3**	>30	*
**4**	>30	*
Hmmthz	>30	*
Hmmthd	>30	*
Hmtt	>30	[64]
[SbCl_3_(Hmtt)_2_]	7.3 ± 0.2	[64]
[SbBr_3_(Hmtt)_2_]	7.2 ± 0.3	[64]
[SbI_3_(Hmtt)_2_]	12.4 ± 0.5	[64]
{[SbCl_2_(Hmtt)_4_]⋅Cl}	9.1 ± 0.5	[64]
{[SbBr_2_(Hmtt)_4_]⋅Br}	15.7 ± 0.5	[64]
MMI	>30	[71]
[BiCl_3_(MMI)_2_]	>30	[71]
[BiBr_3_(MMI)_2_]	>30	[68]
*cisplatin*	3.9 ± 0.1	*
*carboplatin*	23.5 ± 6.4	[70]

* In this study.

**Table 4:** Crystal data and structure refinement results for the complexes.

	1	3	4
**Chemical formula**	C_16_H_20_Br_6_N_4_S_8_Sb_2_	C_8_H_10_BiCl_3_N_2_S_4_	C_6_H_8_BiBr_3_N_4_S_4_
**Formula weight**	1247.80	577.75	713.11
**Crystal system**	monoclinic	monoclinic	triclinic
**Space group**	P2_1_/c	C2/c	P-1
***a*** **, *b*, *c* (Å)**	23.1112(8), 8.1067(2), 20.9778(7)	9.6910(14), 22.303(4), 8.0863(13)	8.6937(6), 10.1525(8), 11.1261(10)
**α, β, γ (°)**	90, 117.003(4), 90	90, 104.923(5), 90	67.443(8), 69.827(8), 69.562(7)
***V*** **(Å^3^)**	3501.8(2)	1688.9(5)	824.17(14)
***Z***	2	4	2
**μ (mm^−1^)**	24.87	11.392	18.462
**No. of measured, independent and** **observed [*I* > 2s(*I*)] reflections**	7553, 6397, 5005	8957, 1545, 1344	5781, 3185, 3058
***R*** **_int_**	0.0449	0.084	0.0269
***R*[*F*^2^ > 2s(*F*^2^)], *wR*(*F*^2^), *S***	0.061, 0.166, 1.03	0.034, 0.077, 1.02	0.0270, 0.0677, 1.128
**No. of reflections**	6397	1545	3185
**No. of parameters**	386	85	166
**Δρ_max_, Δρ_min_ (e Å^−3^)**	1.00, −1.47	1.10, −1.19	1.638, −2.109

## Data Availability

The original contributions presented in this study are included in the article and Supplementary Materials. Crystallographic data for complexes **1**, **3**, and **4** have been deposited with the Cambridge Crystallographic Data Centre (CCDC) under deposition numbers 2534028, 2534029, and 2534027, respectively. Further inquiries can be directed to the corresponding author.

## Supplementary Materials

The following supporting information can be downloaded at: https://www.mdpi.com/article/10.3390/molecules31203783/s1, Figures S1–S6: FT-IR spectra; Figures S7–S10: FT-Raman spectra; Figures S11–S16: ^1^H NMR spectra; Figures S17–S22: ^13^C NMR spectra; Figure S23: UV–Vis spectra; Figures S24 and S25: UV–Vis stability studies in DMSO and PBS, respectively; Figures S26–S29: TG–DTA curves; Figure S30: dose–response curves of 1–2 (A), 3–4 (B), and Hmmthd and Hmmthz (C) in the antiproliferative studies against HeLa cells; Table S1: geometrical parameters of hydrogen-bonding interactions; Table S2: molar conductivity data.
