# Supplementary material for: Antimony(III) and Bismuth(III) Halide Complexes Containing Thiazole-and Thiadiazole-Based Mercapto Ligands: Synthesis, Structural Characterization, and Biological Activity
Source: Molecules. 2026 Oct 21;31(20):3783. doi: 10.3390/molecules31203783 (PMC13707501; doi:10.3390/molecules31203783)
Supplement: Supplementary file 1 [file molecules-31-03783-s001.zip › Suplemantary Materials.pdf]

# Antimony(III) and bismuth(III) halide complexes containing thiazole- and thiadiazole-based mercapto ligands: Synthesis, structural characterization and biological activity

Ibrahim I. Ozturk, Cemile Albayrak, Anita M. Grześkiewicz, Maciej Kubicki, Christina N. Banti, Sotiris K. Hadjikakou

S1.FT-IR spectra (Figures S1-S6)………………………………………………………...……2

S2. FT-Raman spectra (Figures S7-S10)……………………………………………..…...5

S3. $^{1}$H-NMR spectra (Figures S11-S16)……………………………………………..……7

S4. $^{13}$C-NMR spectra (Figures S17-S22)……………………………………..……….10

S5. UV-Vis spectra (Figure S23)……………………………………………………….…...13

S6. UV-Vis stability in DMSO (0-48h) (Figure S24)……………………………………….13

S7. UV-Vis stability in PBS (0-48h) (Figure S25)………………………………………….14

S8. TG-DTA curves (Figures S26-S29)…………………………………………………….14

S9. Geometrical parameters of hydrogen-bonding interactions (D–H···A) for the antimony(III) and bismuth(III) complexes (Table S1)…..……………………..…………………………17

S10. Molar conductivity table (Table S2)………………………………………..………18

S11.Dose-response curves of **1-2** (A), **3-4** (B) and Hmmthd, Hmmthz (C) for the antiproliferative studies towards HeLa cells (Figure S30)…………………………………….19

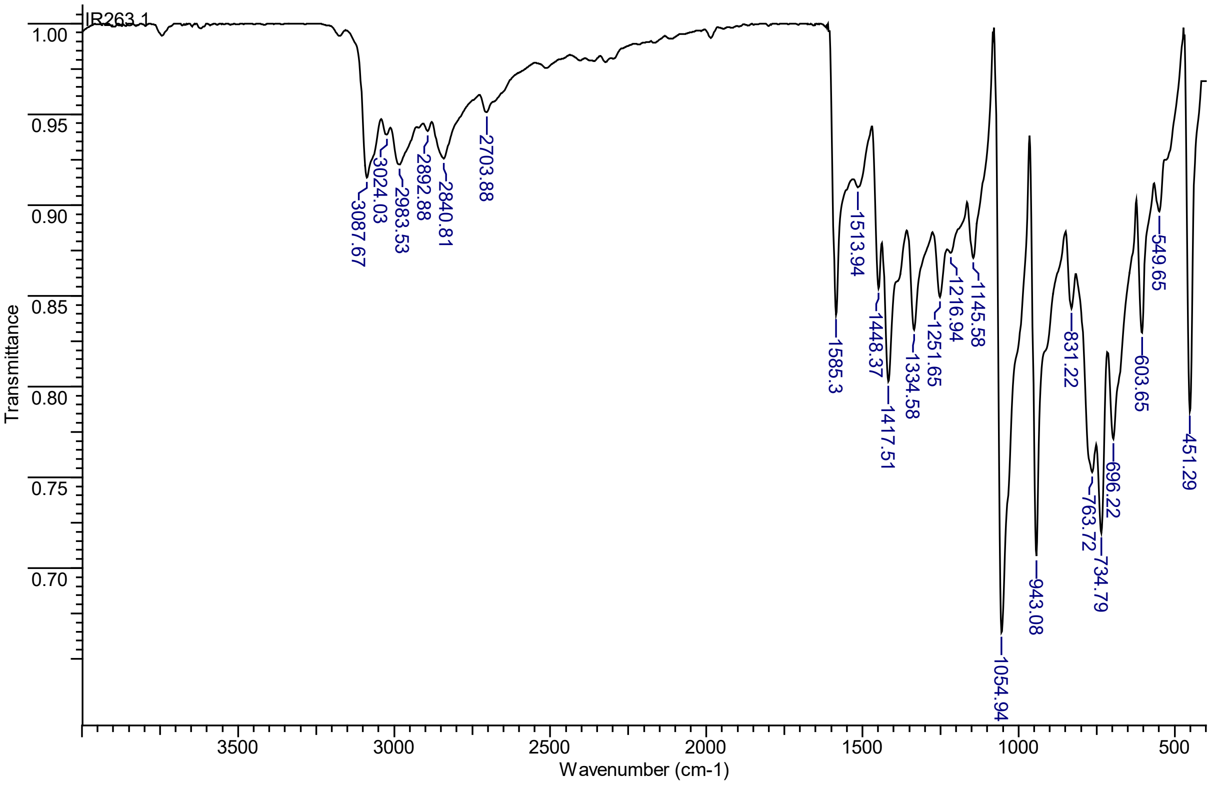


**Fig. S1.** FT-IR spectrum of Sb(Hmmthz)$_2$Br$_3$ (**1**).

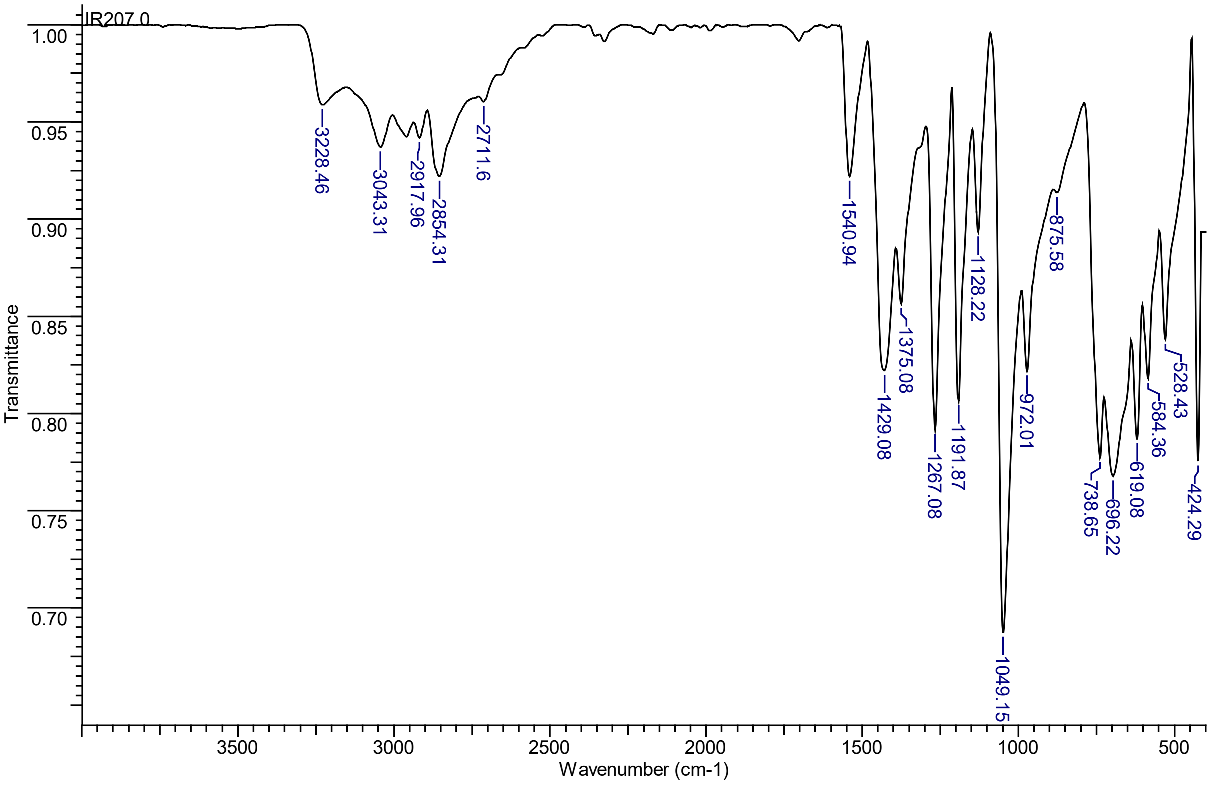


**Fig. S2.** FT-IR spectrum of Sb(Hmmthd)$_3$Br$_3$ (**2**).

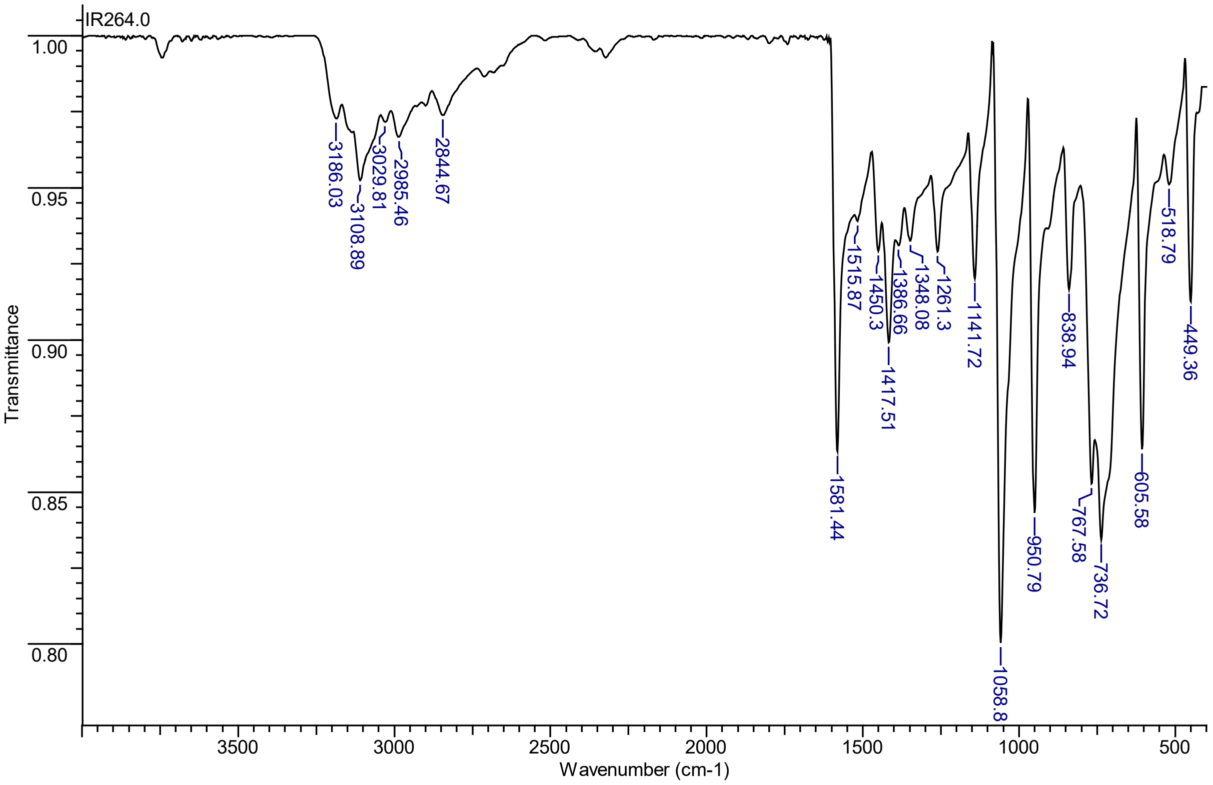


**Fig. S3.** FT-IR spectrum of $Bi(Hmmthz)_2Cl_3$ (**3**).

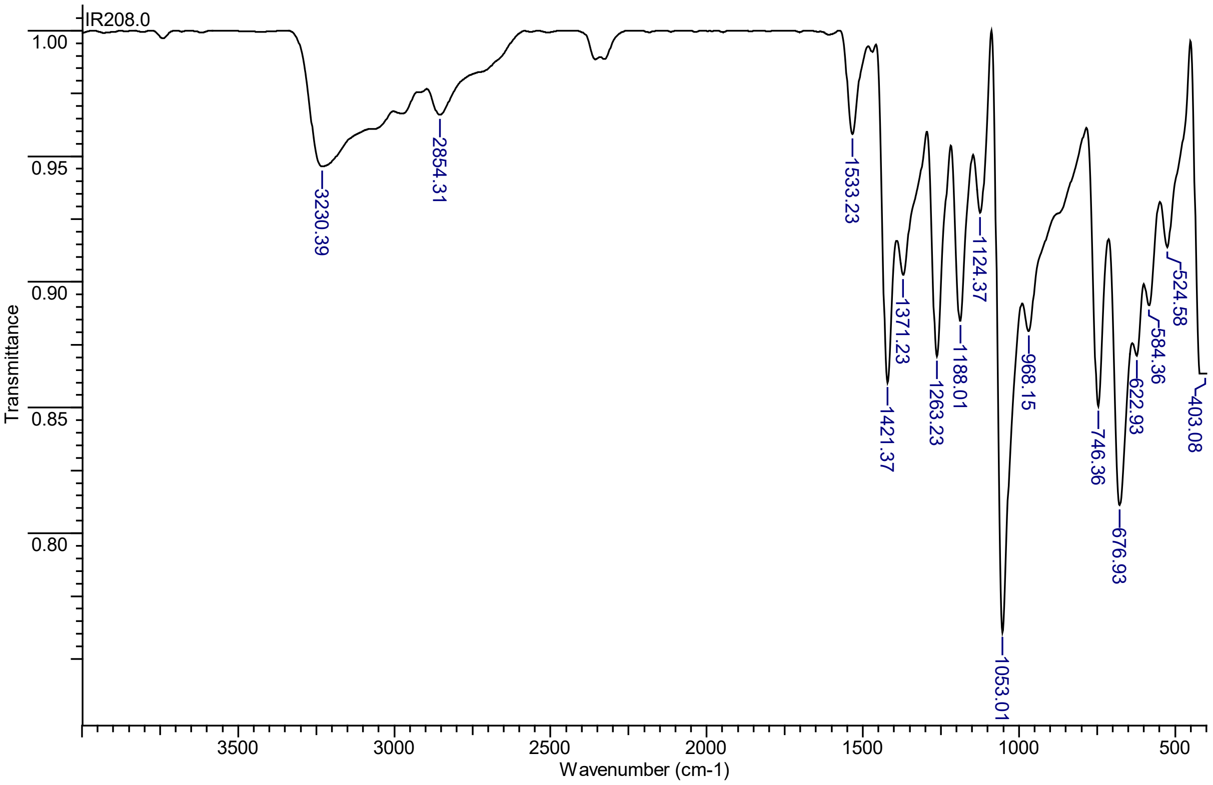


**Fig. S4.** FT-IR spectrum of $Bi(Hmmthd)_3Br_3$ (**4**).

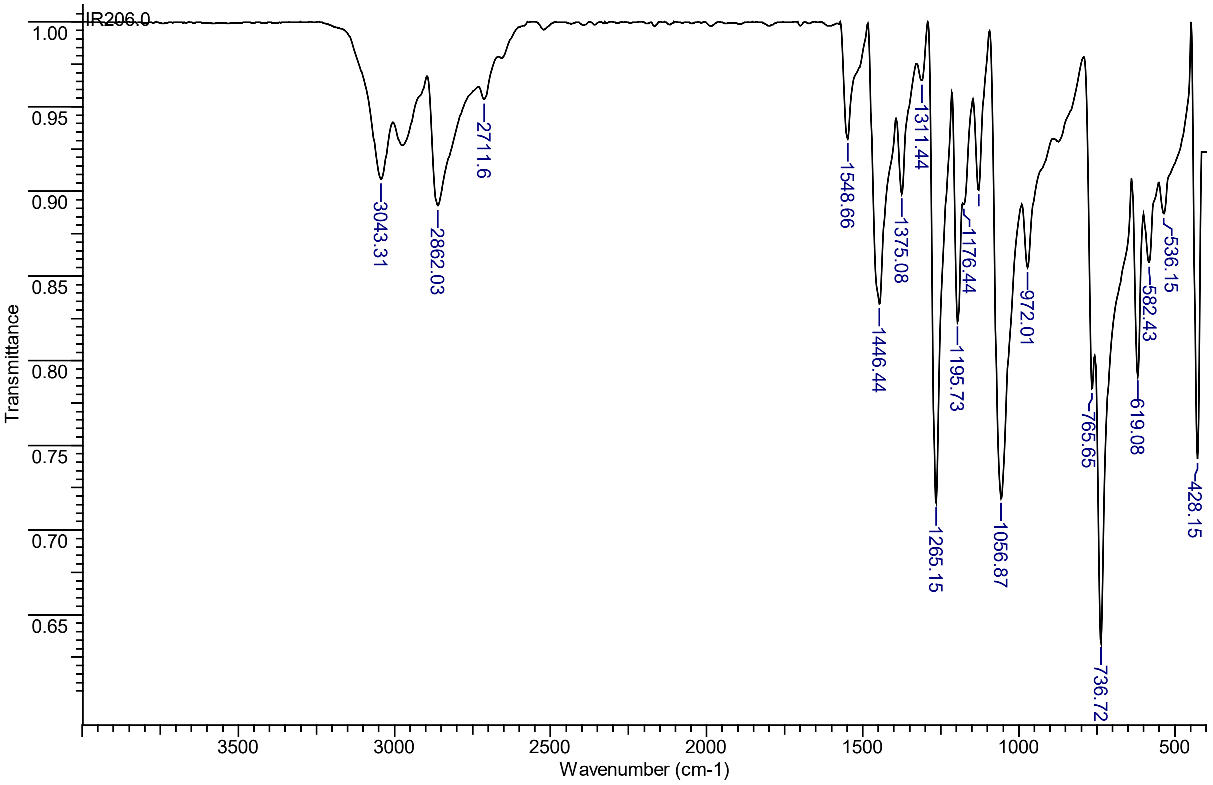


**Fig. S5.** FT-IR spectrum of 2-mercapto-5-methyl-1,3,4-thiadiazole (Hmmthd).

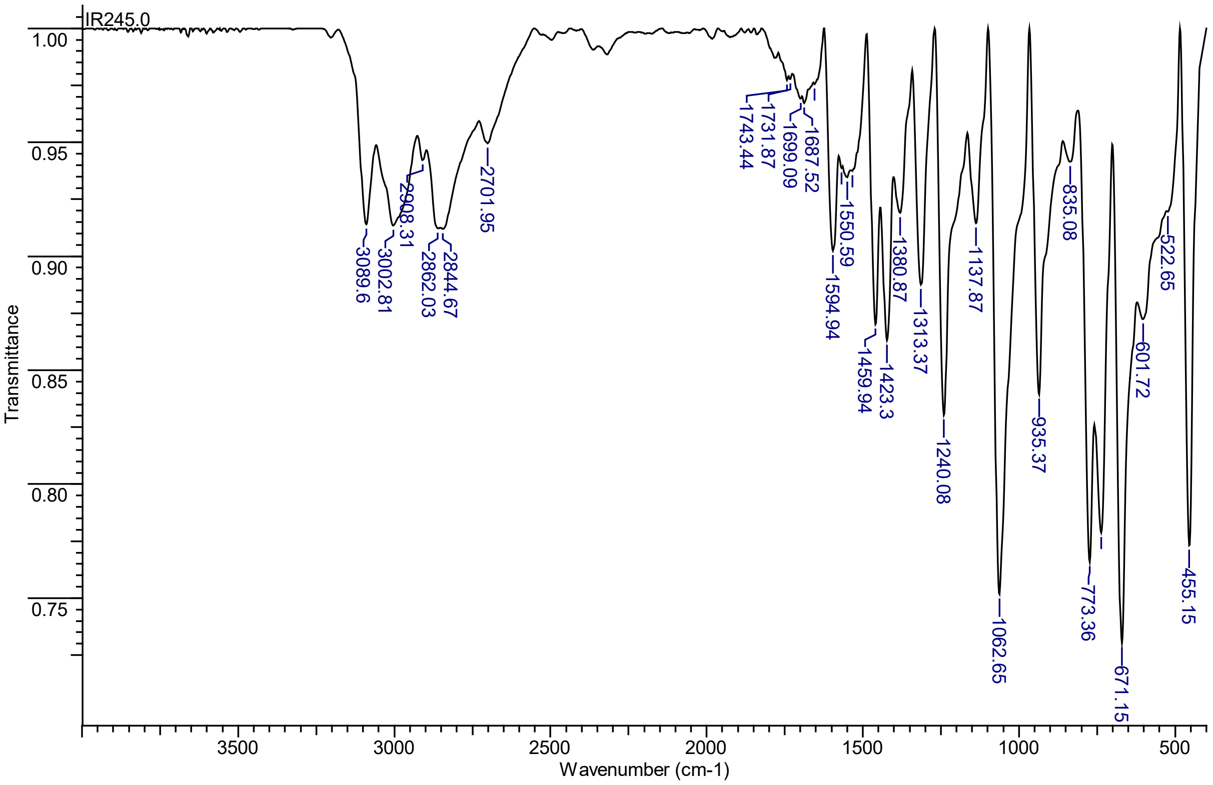


**Fig. S6.** FT-IR spectrum of 2-mercapto-4-methylthiazole (Hmmthz).

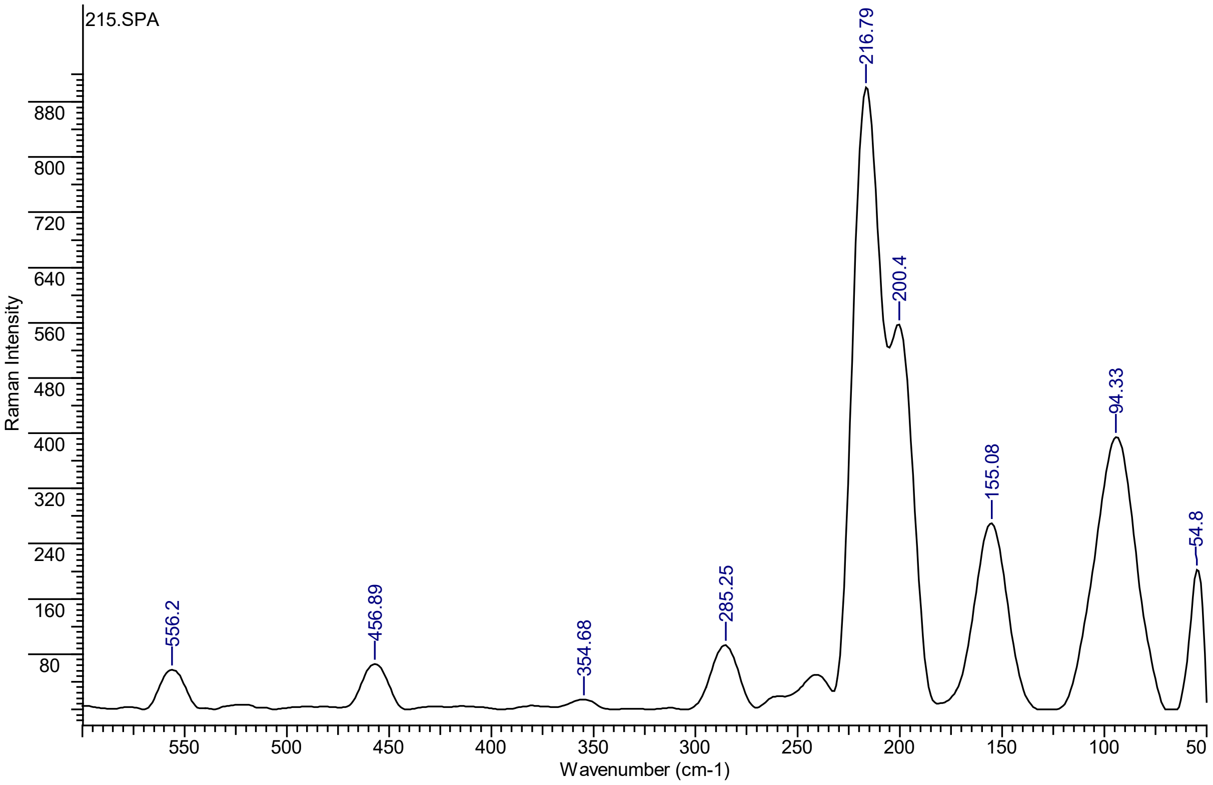


**Fig. S7.** Raman spectrum of $Sb(Hmmthz)_2Br_3$ (**1**).

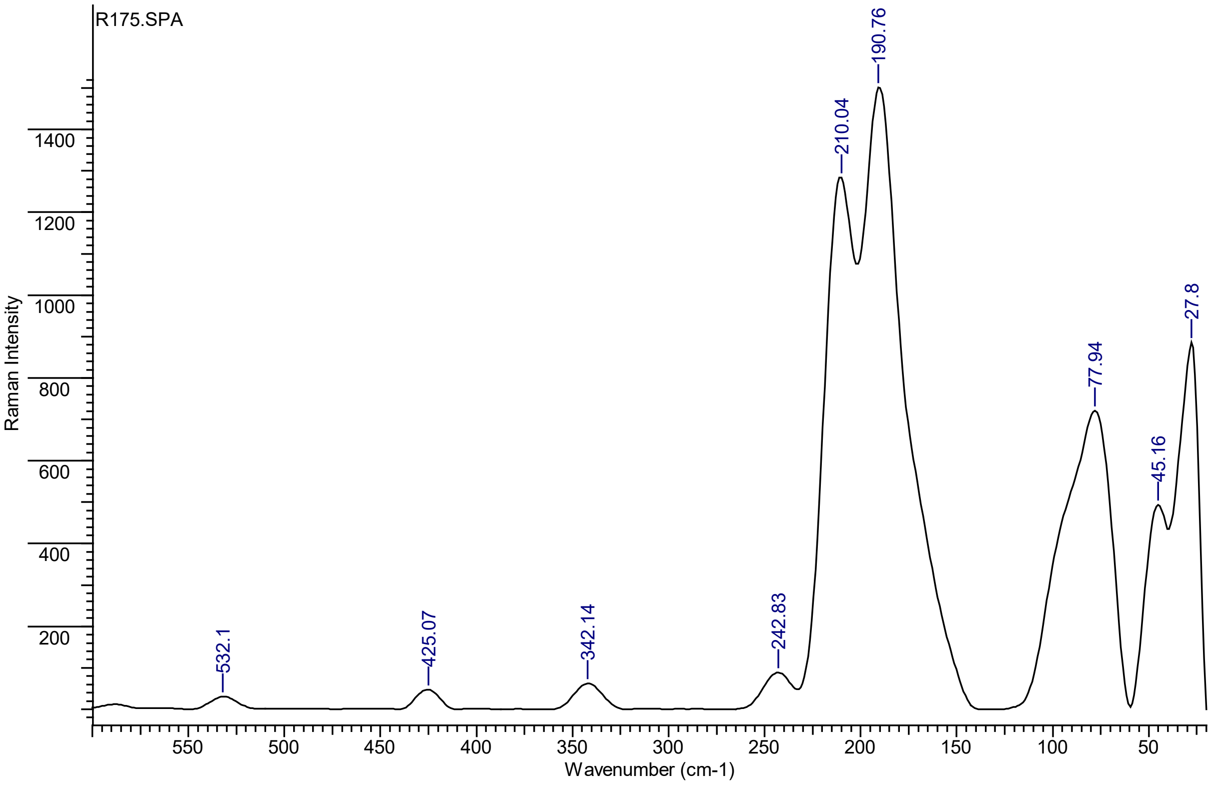


**Fig. S8.** Raman spectrum of $Sb(Hmmthd)_3Br_3$ (**2**).

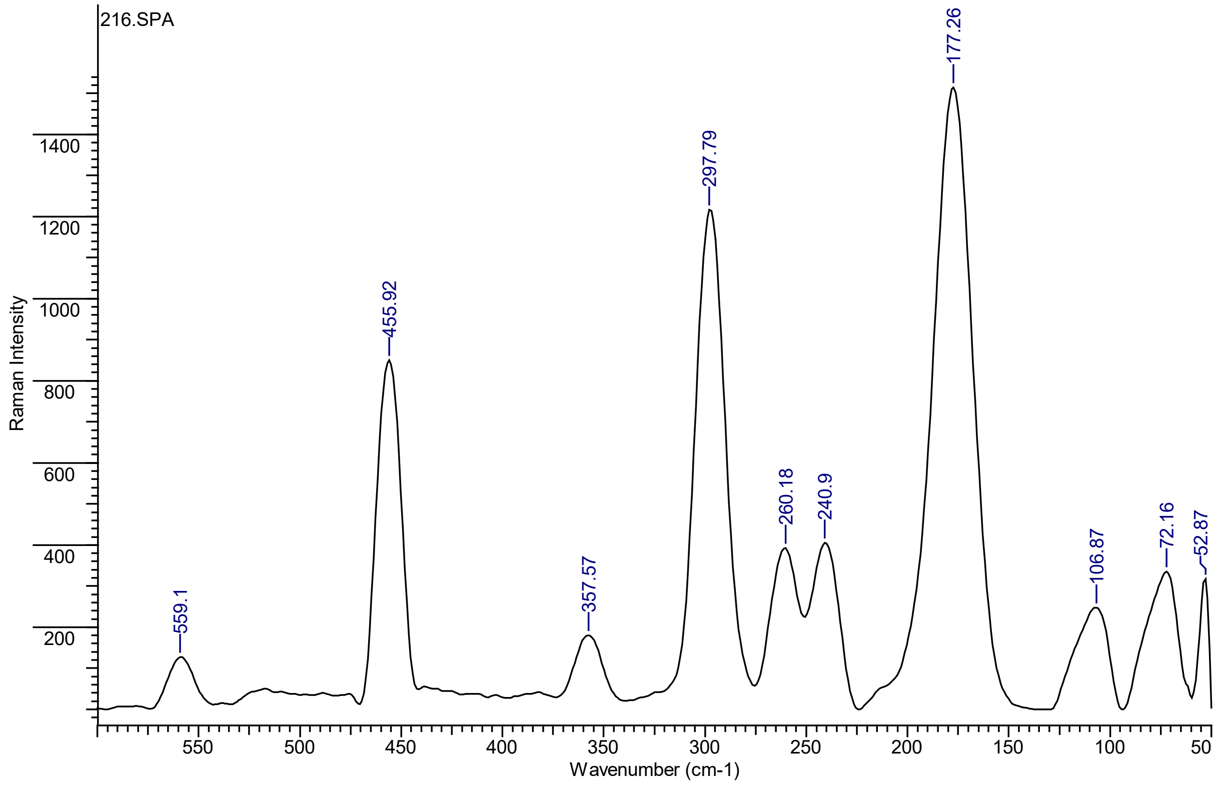


**Fig. S9.** Raman spectrum of Bi(Hmmthz)$_2$Cl$_3$ (**3**).

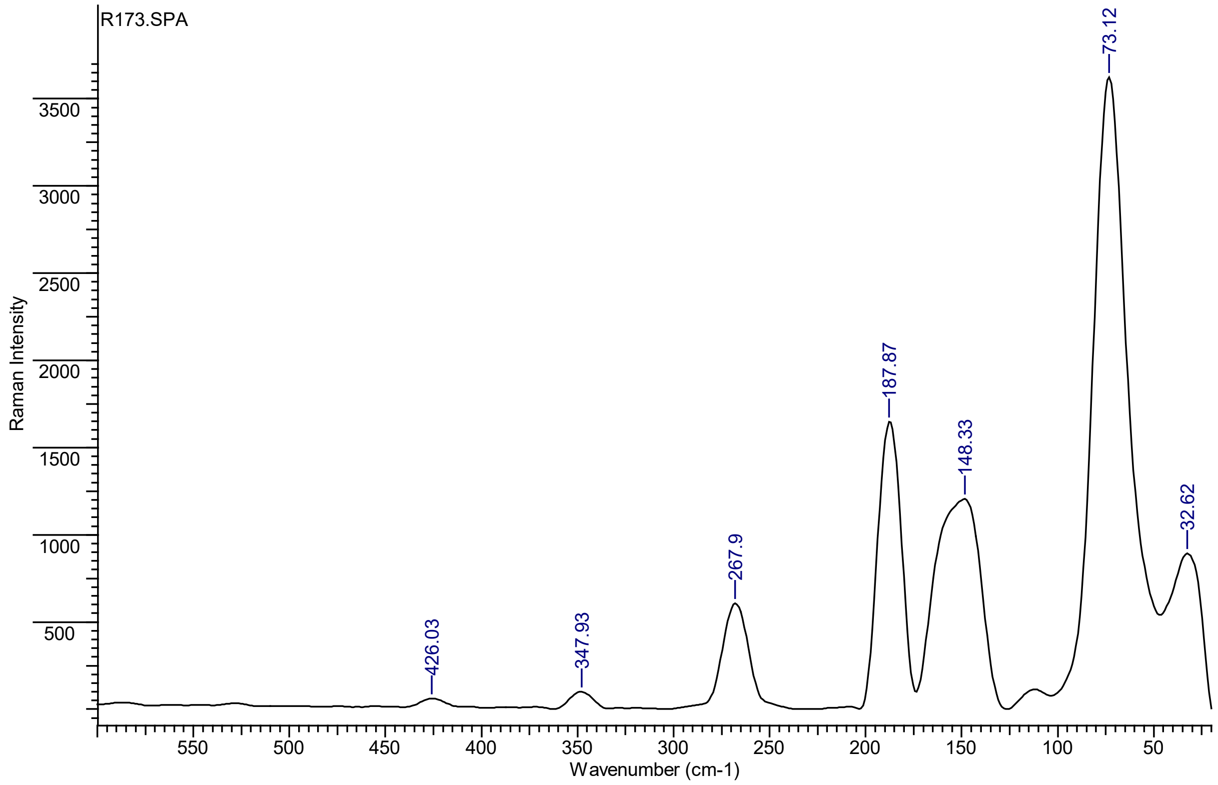


**Fig. S10.** Raman spectrum of Bi(Hmmthd)$_3$Br$_3$ (**4**).

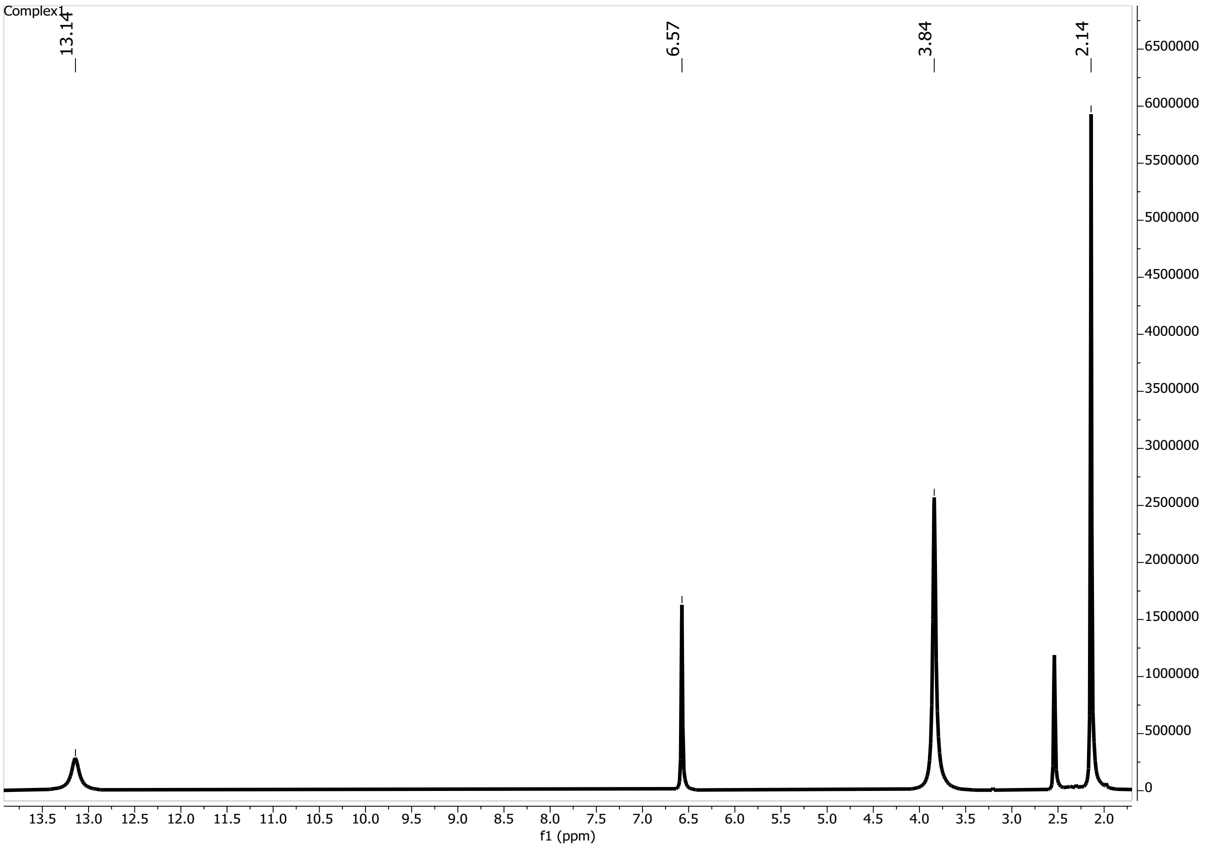


**Fig. S11.** $^{1}$H-NMR spectrum of Sb(Hmmthz)$_2$Br$_3$ (**1**).

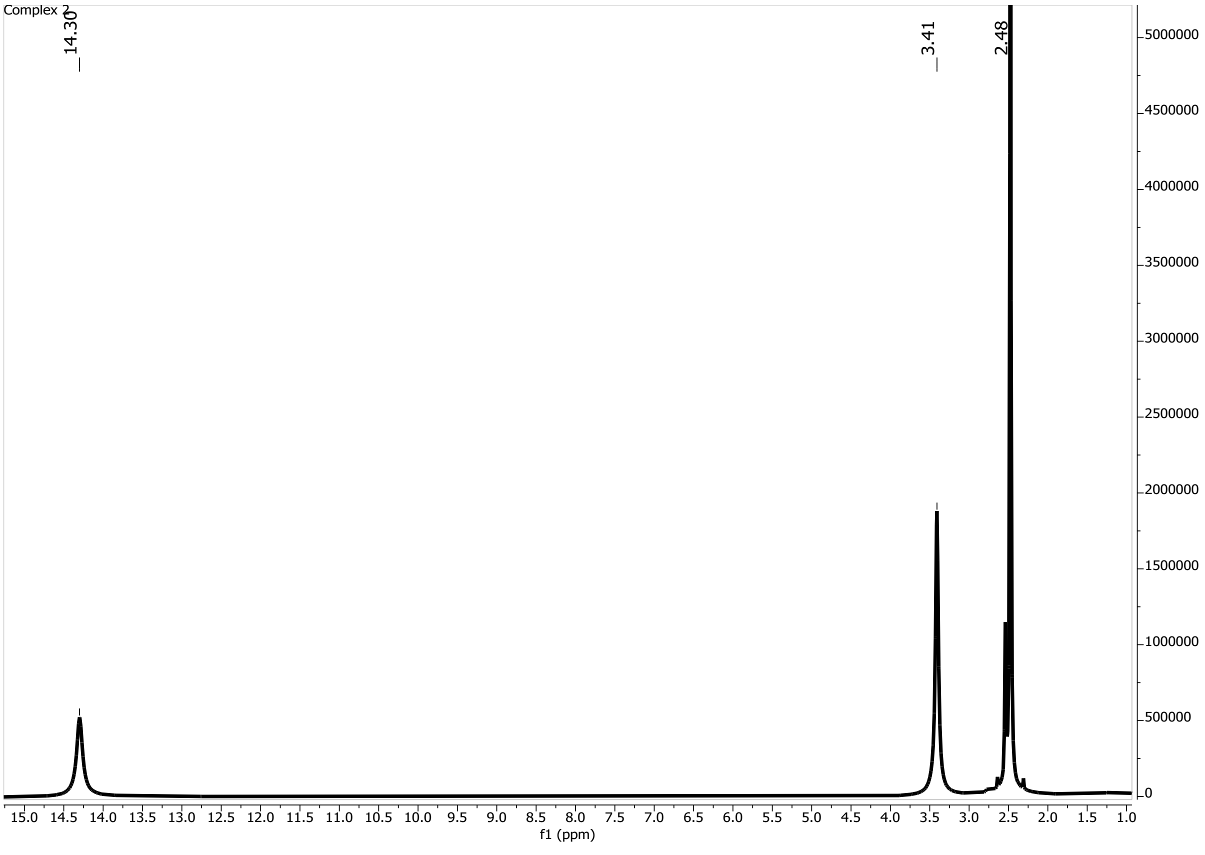


**Fig. S12.** $^{1}$H-NMR spectrum of Sb(Hmmthd)$_3$Br$_3$ (**2**).

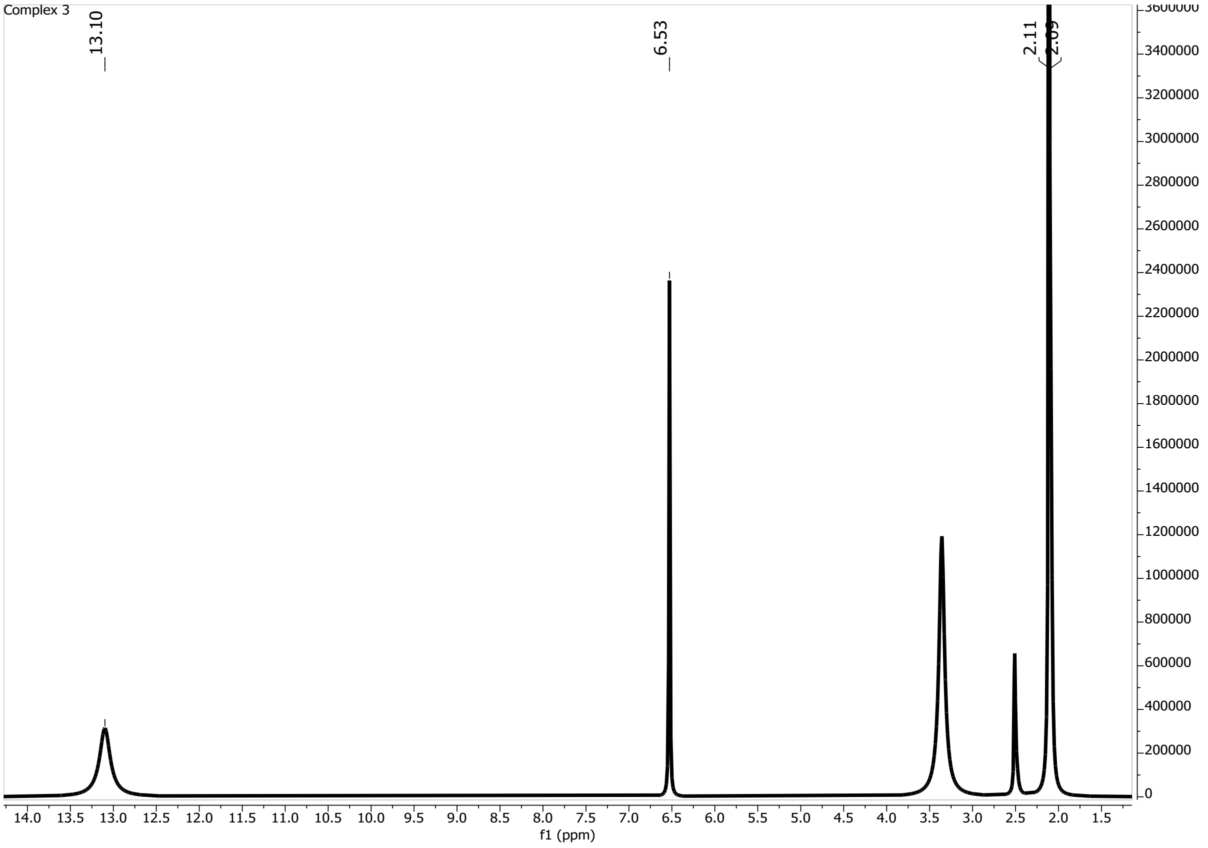


**Fig. S13.** $^{1}$H-NMR spectrum of Bi(Hmmthz)$_2$Cl$_3$ (**3**).

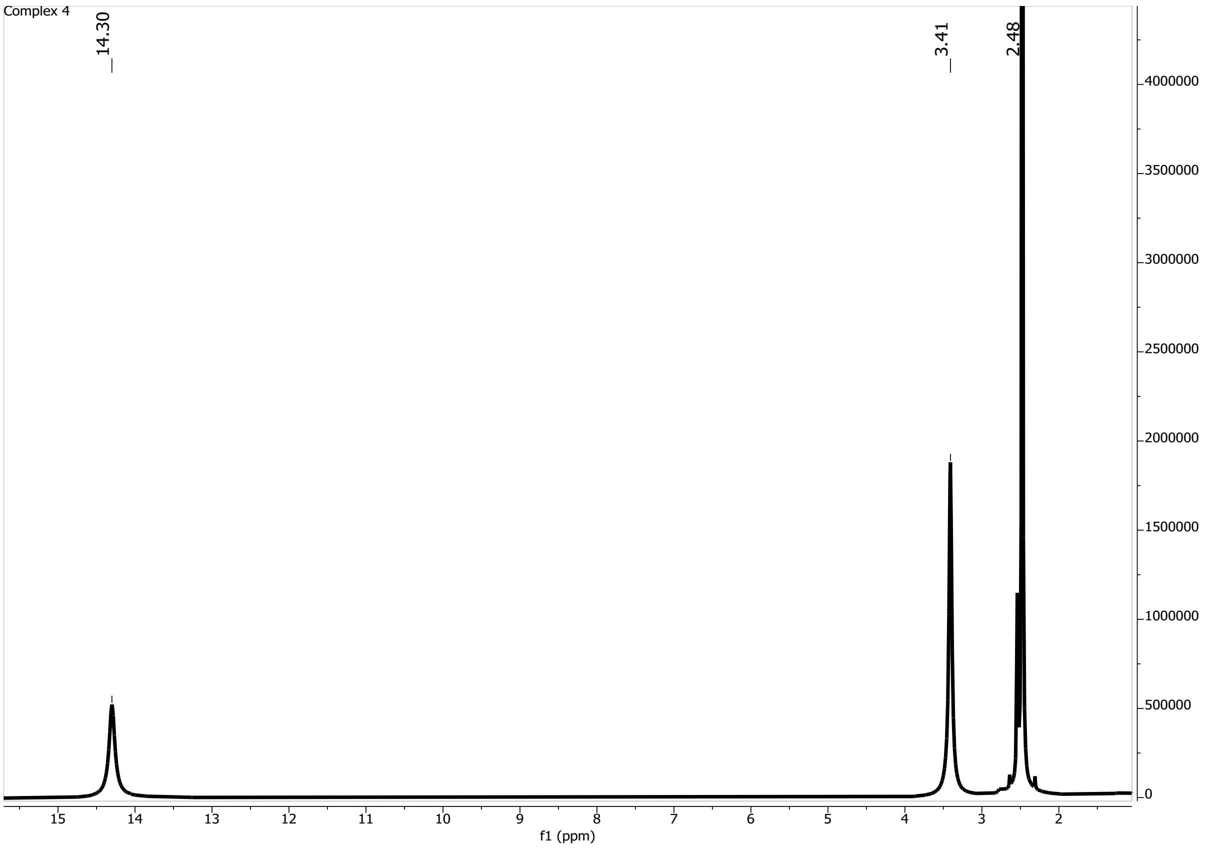


**Fig. S14.** $^{1}$H-NMR spectrum of Bi(Hmmthd)$_3$Br$_3$ (**4**).

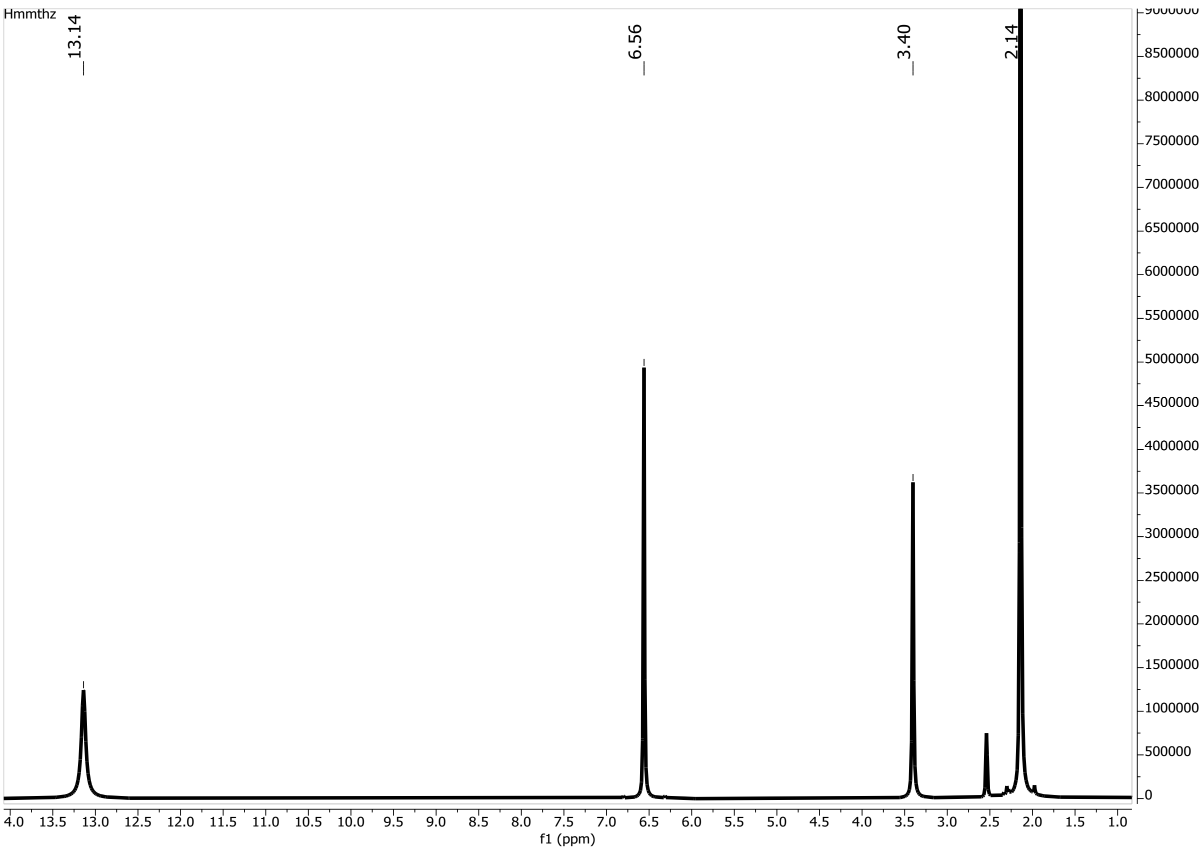


**Fig. S15.** $^{1}$H-NMR spectrum of 2-mercapto-4-methylthiazole (Hmmthz).

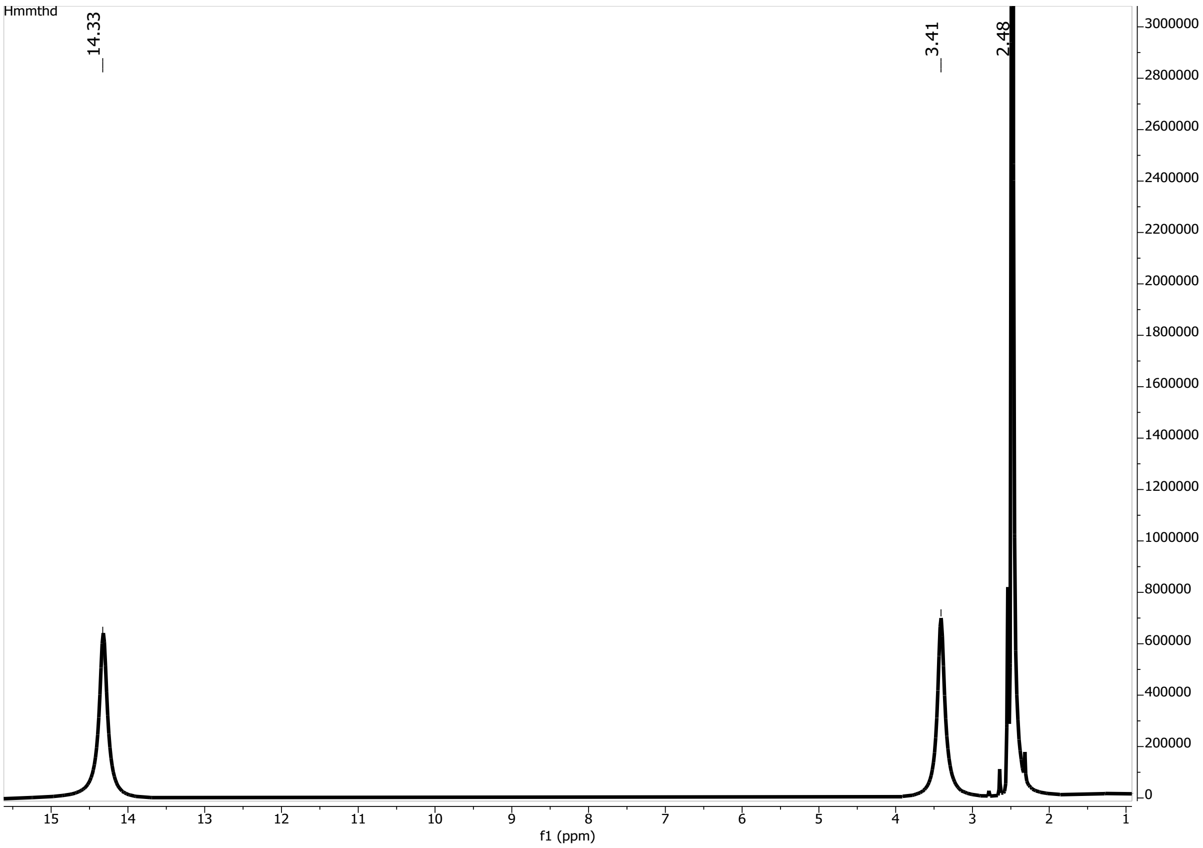


**Fig. S16.** $^{1}$H-NMR spectrum of 2-mercapto-5-methyl-1,3,4-thiadiazole (Hmmthd).

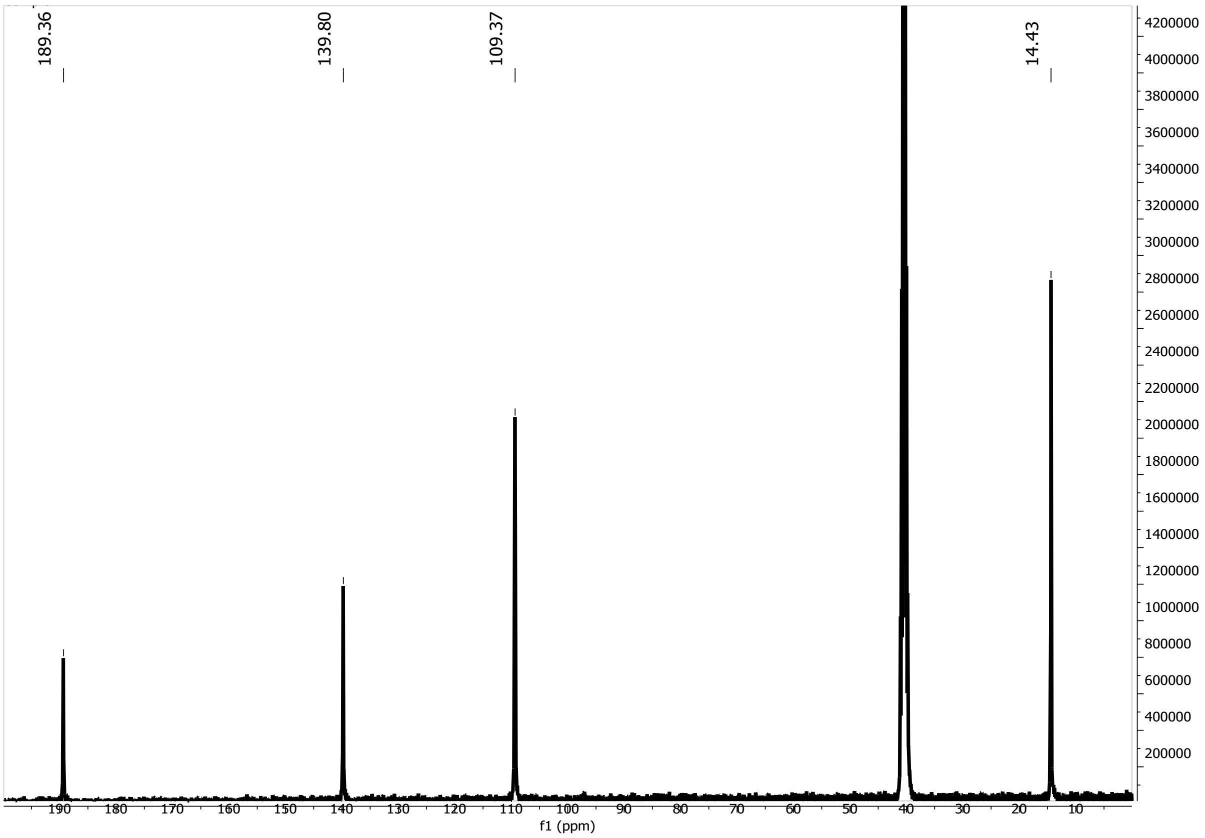


**Fig. S17.** $^{13}$C-NMR spectrum of $Sb(Hmmthz)_2Br_3$ (**1**).

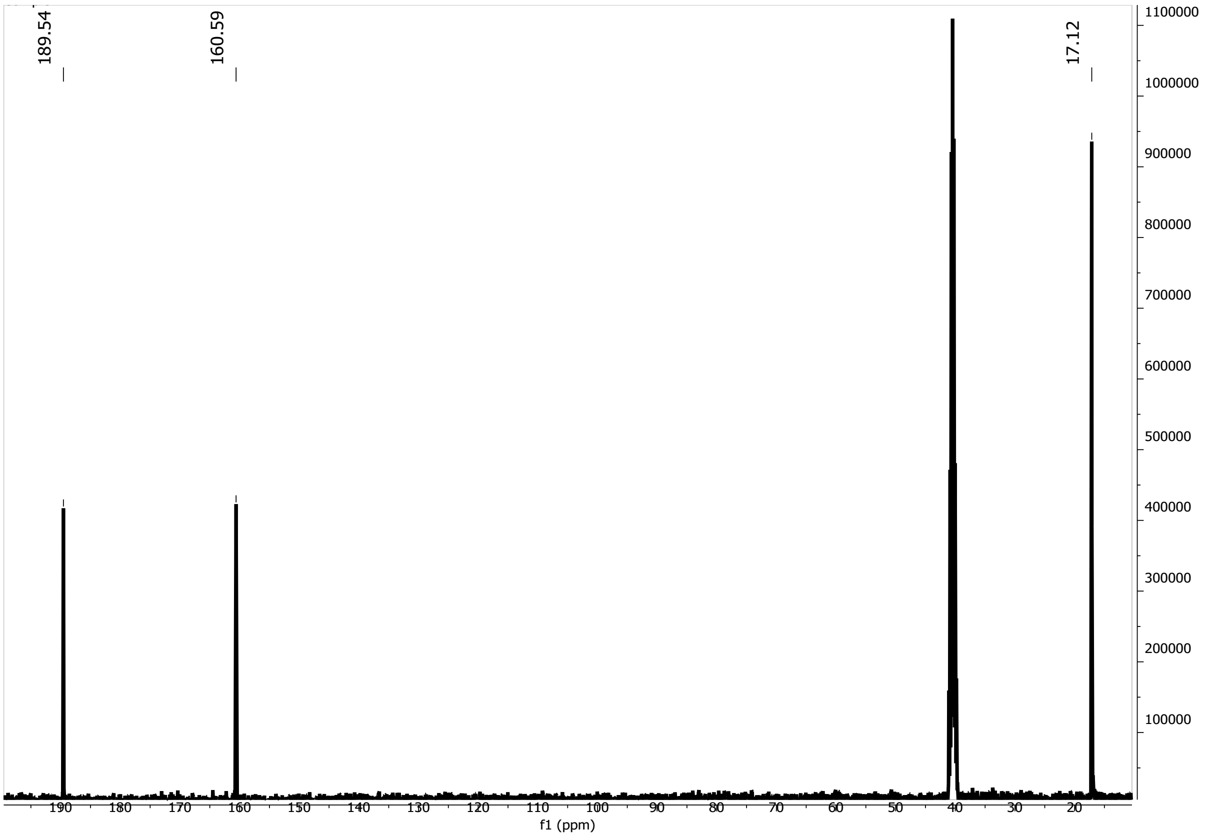


**Fig. S18.** $^{13}$C-NMR spectrum of $Sb(Hmmthd)_3Br_3$ (**2**).

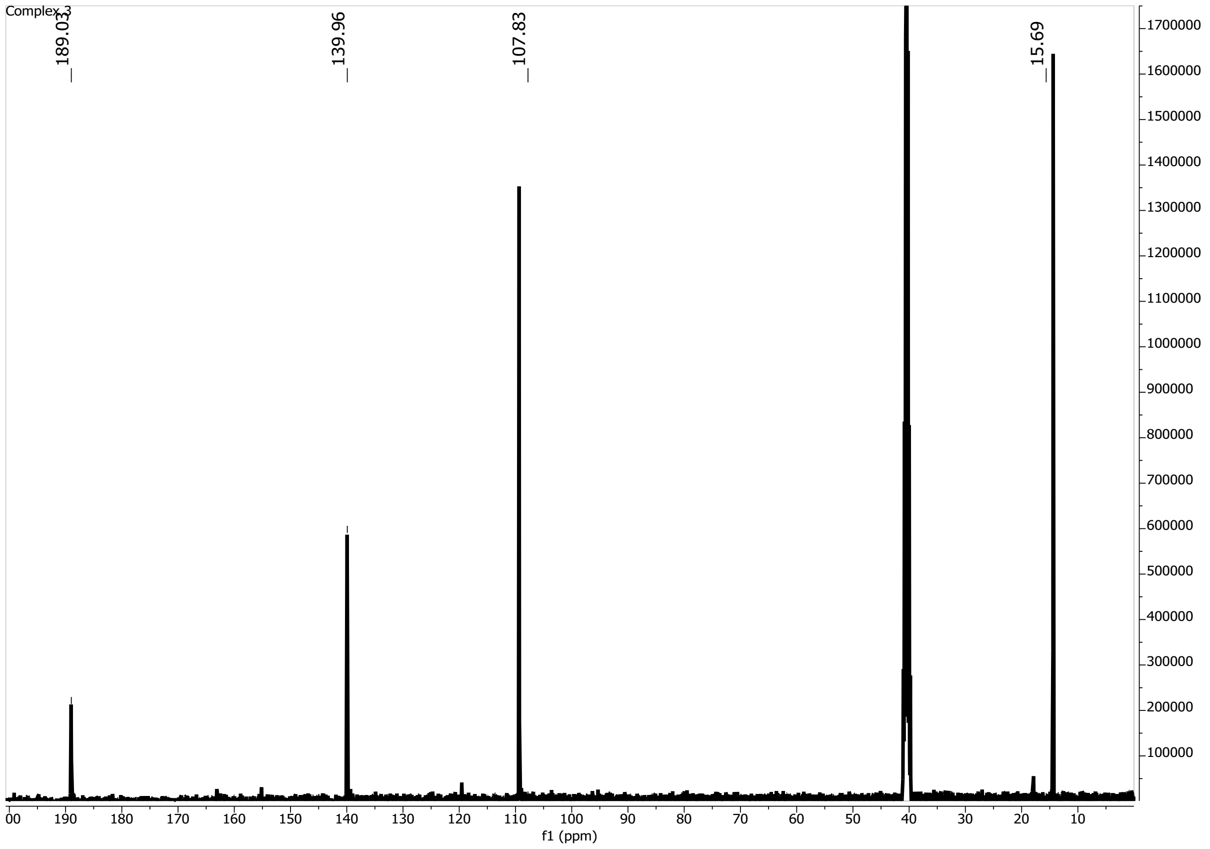


**Fig. S19.** $^{13}C$-NMR spectrum of Bi(Hmmthz)$_2$Cl$_3$ (**3**).

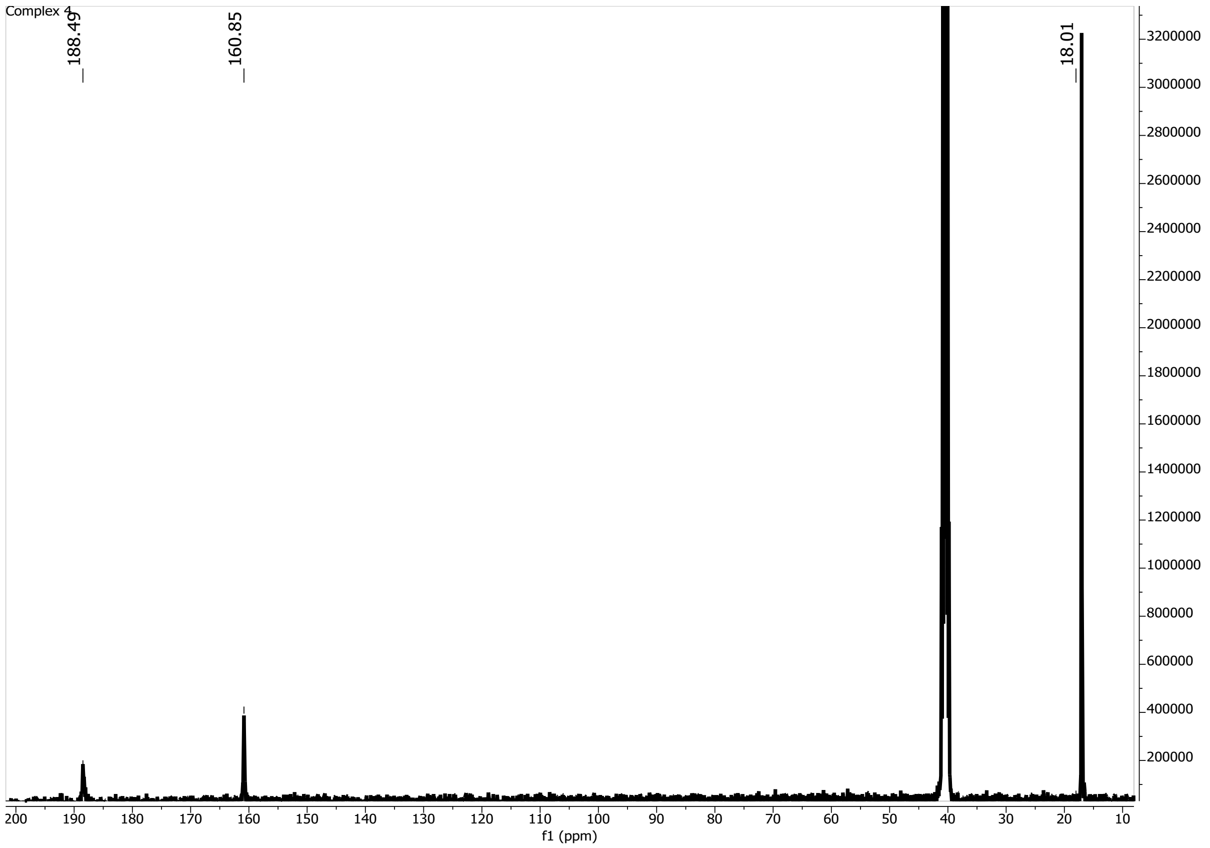


**Fig. S20.** $^{13}C$-NMR spectrum of Bi(Hmmthd)$_3$Br$_3$ (**4**).

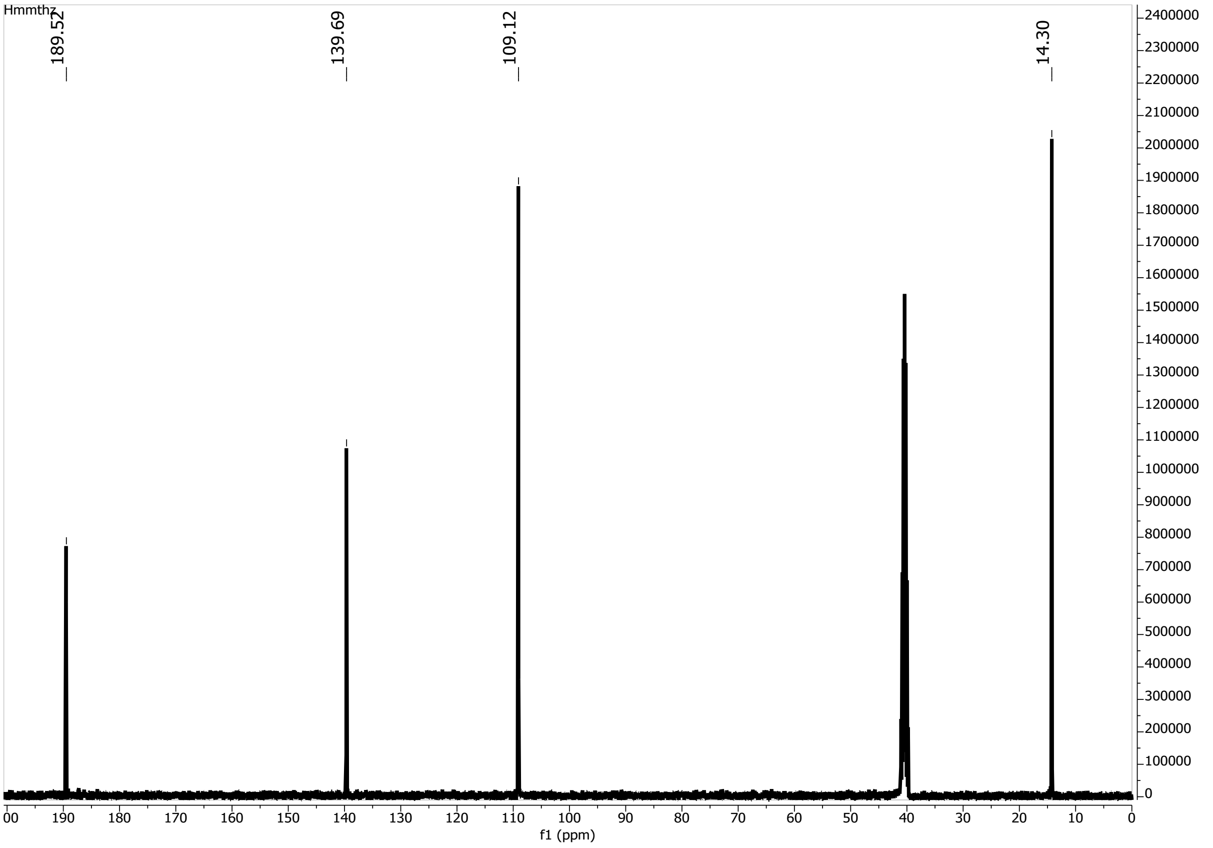


**Fig. S21.** $^{13}$C-NMR spectrum of 2-mercapto-4-methylthiazole (Hmmthz).

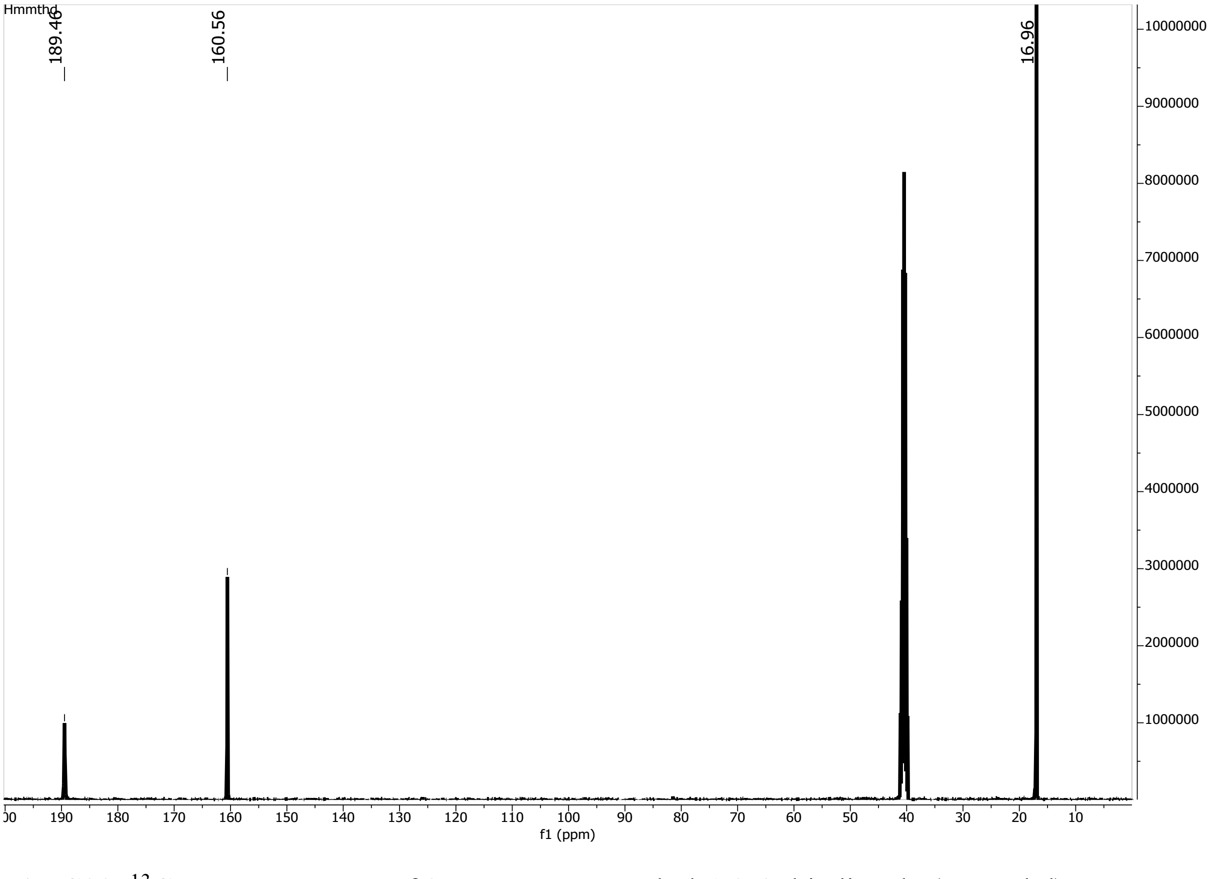


**Fig. S22.** $^{13}$C-NMR spectrum of 2-mercapto-5-methyl-1,3,4-thiadiazole (Hmmthd).

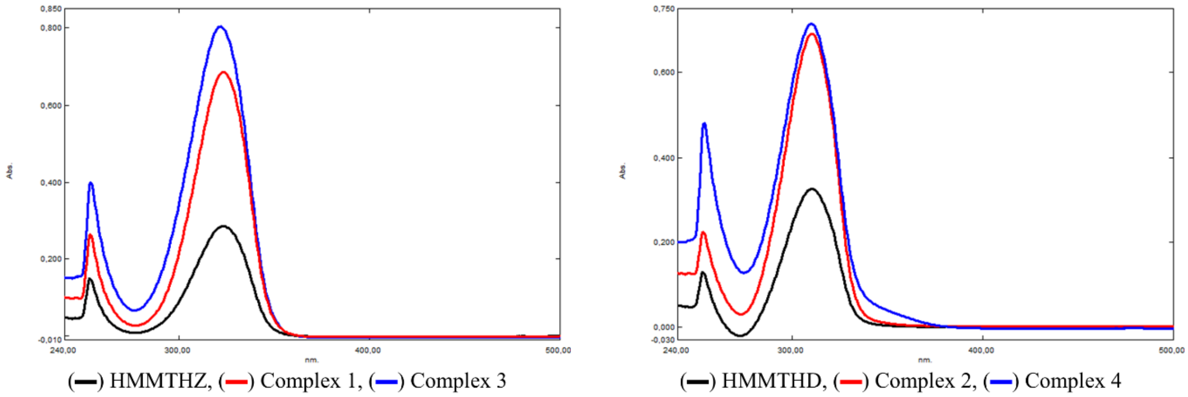


**Fig. S23.** UV spectra of antimony (III) and bismuth(III) complexes and their free ligands ($2x10^{-5}$ M)

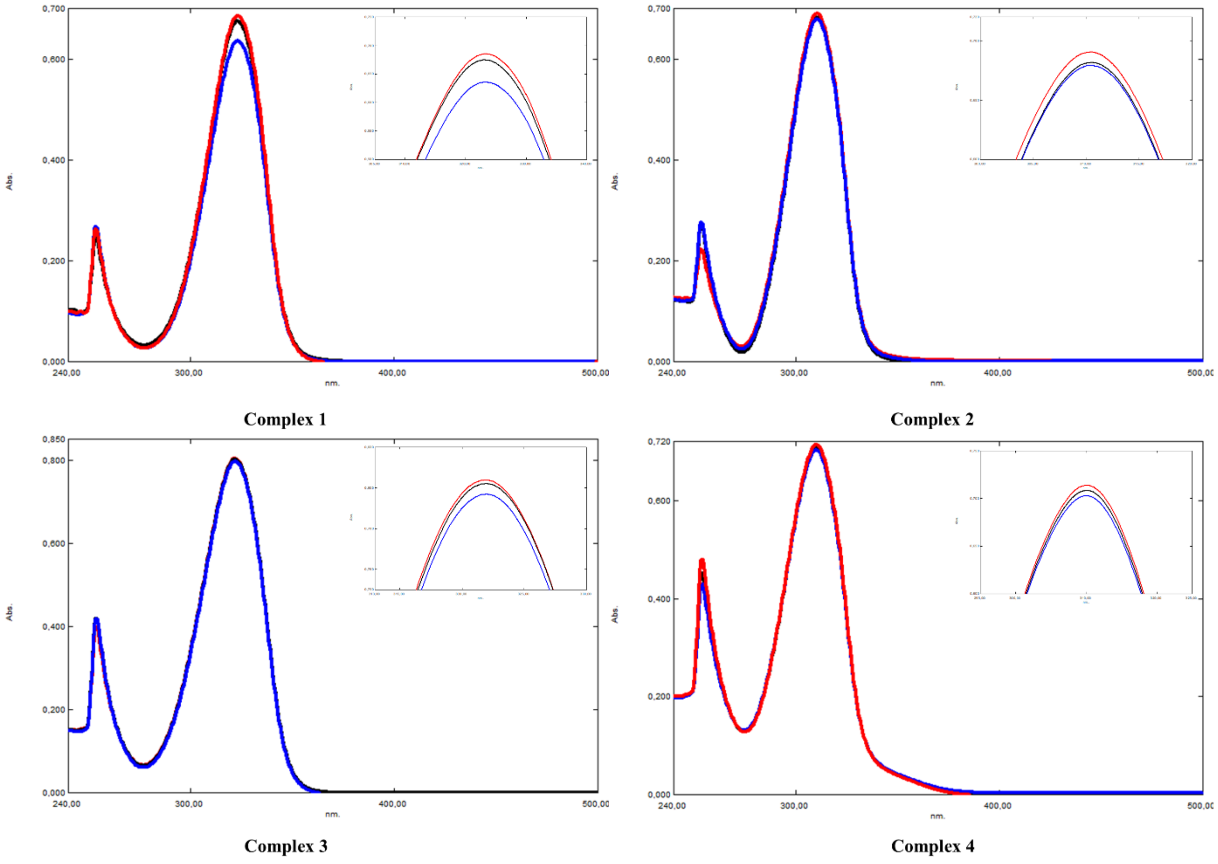


**Fig. S24.** UV spectra of the antimony (III) and bismuth(III) complexes for 0 h (—), 24 h (—) and 48 h (—) in DMSO solution ($2x10^{-5}$ M).

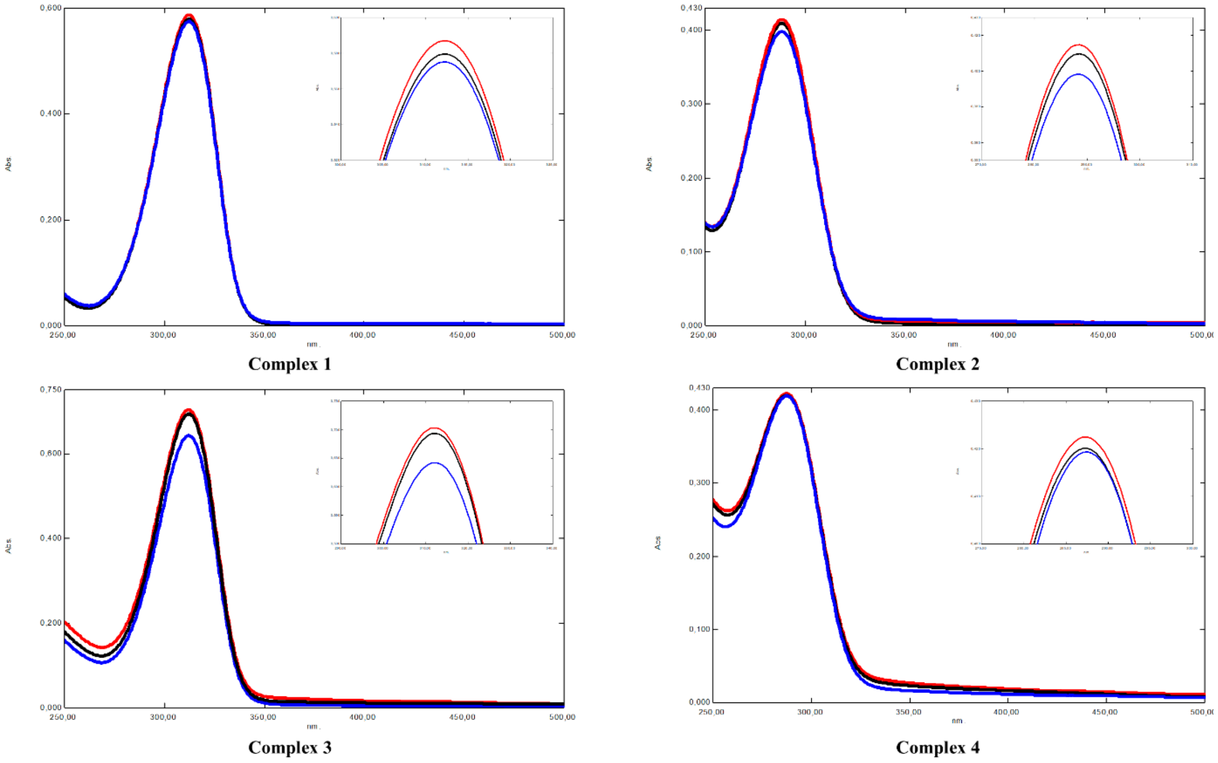


**Fig. S25.** UV spectra of the antimony (III) and bismuth(III) complexes for 0 h (▬), 24 h (▬) and 48 h (▬) in PBS (2x$10^{-5}$ M).

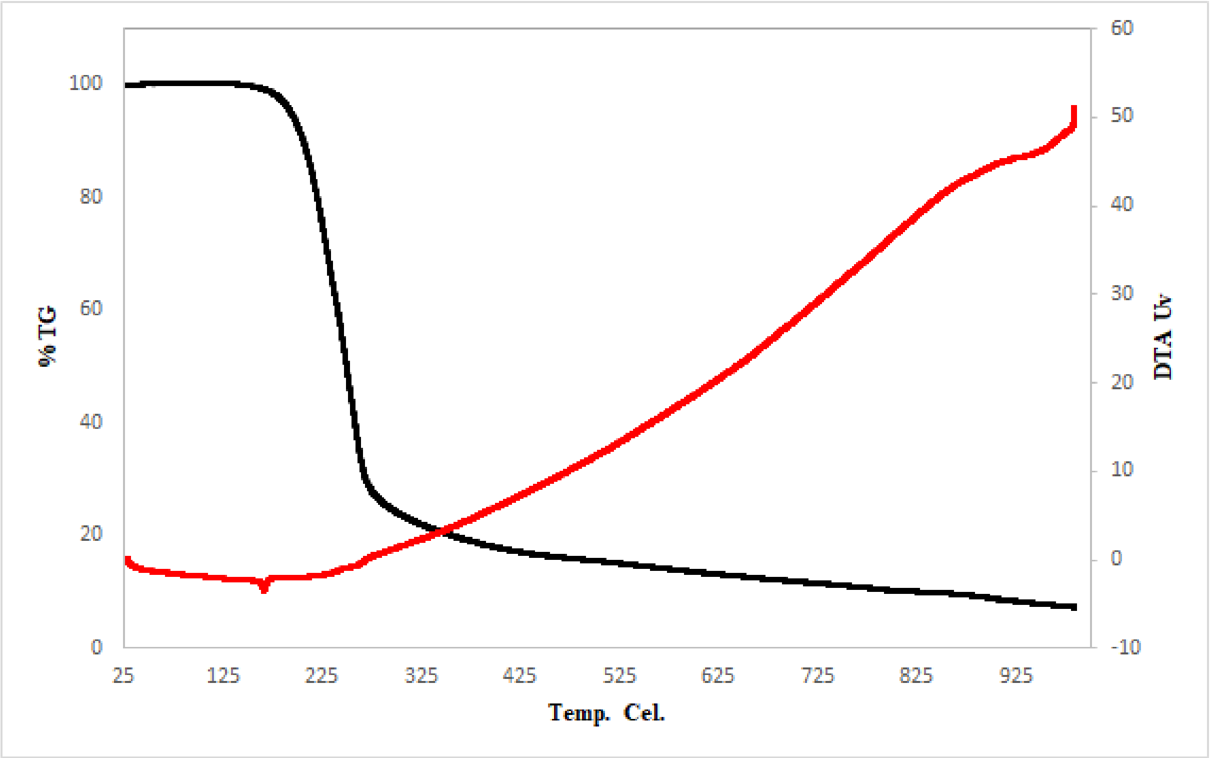


**Fig. S26.** TG–DTA curves of [{$SbBr_2(\mu_2\text{-}Br)(Hmmthz)_2\}_n$] (**1**).

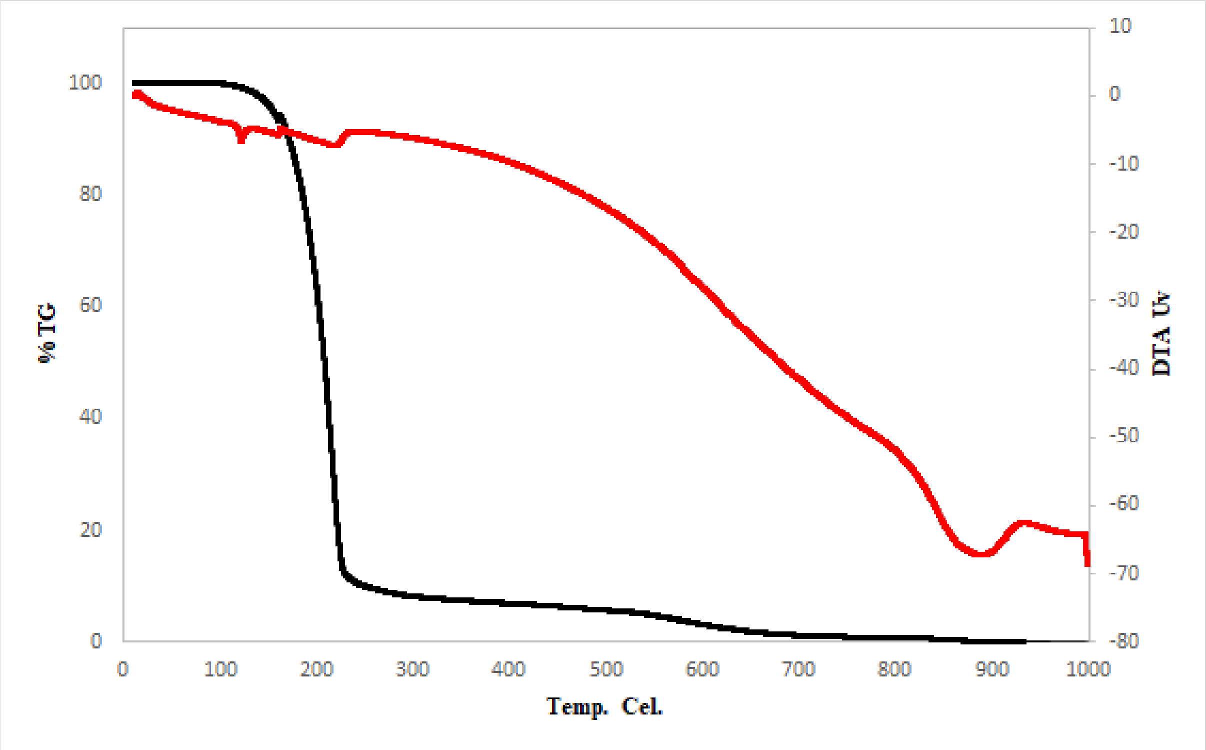


**Fig. S27.** TG–DTA curves of [{$SbBr_2$($\mu_2$-Br($Hmmthd)_2$}$_2$] (**2**).

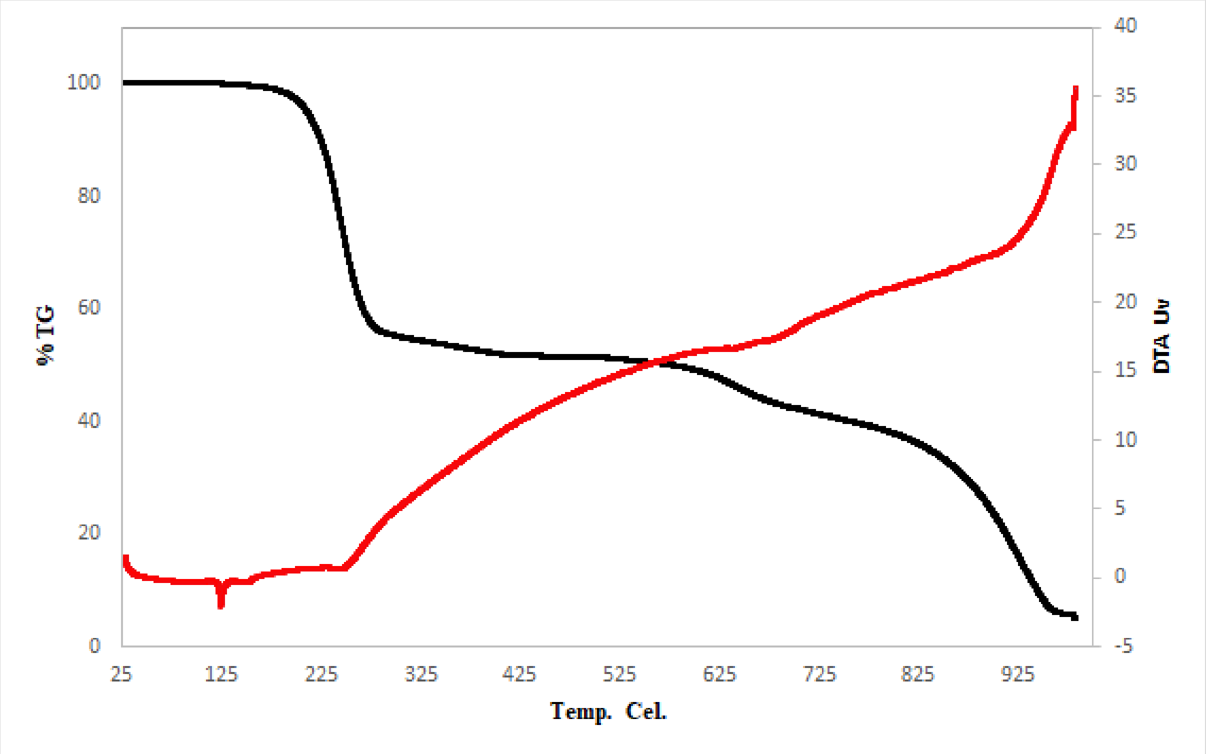


**Fig. S28.** TG–DTA curves of [{$BiCl_2$($\mu_2$-Cl)($Hmmthz)_2$}$_n$] (**3**).

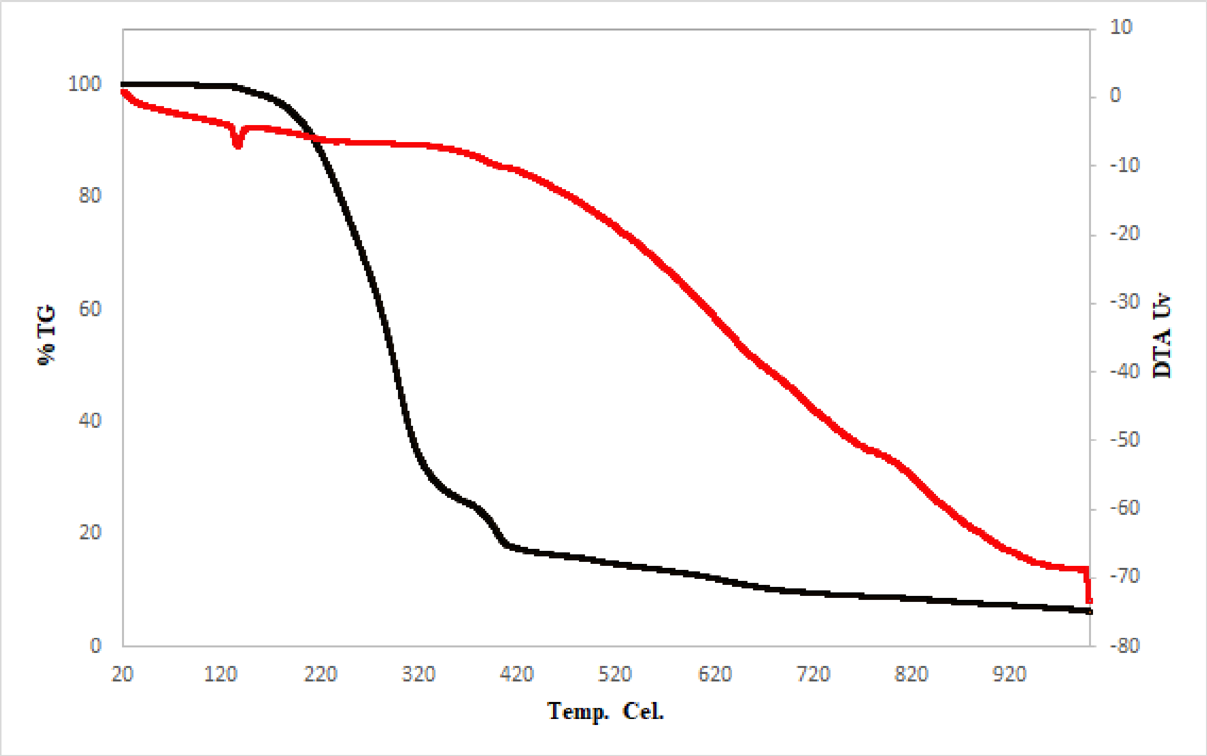


**Fig. S29.** TG–DTA curves of [{$BiBr_2$($\mu_2$-Br(Hmmthd)$_2$}$_2$] (**4**).

**Table S1.** Geometrical parameters of hydrogen-bonding interactions (D–H···A) for the complexes 1 and 4.

| Donor-H···Acceptor | D-H (Å) | H…A (Å) | D…A (Å) | D-H…A (°) | Symmetry Codes* |
|---|---|---|---|---|---|
| | | | **1** | | |
| N3-H3…Br3 | 0.88 | 2.503 | 3.38(2) | 176 | |
| N33-H33…Br6 | 0.88 | 2.458 | 3.33(1) | 172 | |
| N13-H13…Br3 | 0.88 | 2.489 | 3.35(2) | 167 | |
| N23-H23…Br6 | 0.88 | 2.528 | 3.38(1) | 165 | |
| | | | **4** | | |
| N3-H3…Br3* | 0.880 | 2.7372 | 3.450(6) | 139.0 | -1+x, y, z |
| C5’-H5’A…Br1* | 0.980 | 3.0469 | 3.893(5) | 145.3 | -1+x, y, 1+z |
| C5’-H5’B…Br1* | 0.981 | 3.0040 | 3.711(5) | 130.0 | -x, 1-y, 1-z |
| N13-H13…Br1* | 0.880 | 2.6602 | 3.380(5) | 139.7 | 1+x, y, z |
| C15’-H15C…Br3* | 0.980 | 2.8627 | 3.726(7) | 147.3 | 1-x, 1-y, 1-z |
| C15’-H15A…Br1* | 0.980 | 3.0143 | 3.724(8) | 130.3 | 1-x, 1-y, -z |

**Table S2.** Molar conductivity values of the antimony(III) and bismuth(III) halide complexes for 0 h, 24 h and 48 h in dimethyl sulfoxyde (DMSO) and dimethyl formamide (DMF) ($\Omega^{-1}cm^2mol^{-1}$).

| **Complexes** | **DMF** | **DMSO** | | |
|---|---|---|---|---|
| | | **0 h** | **24 h** | **48 h** |
| **1** | 6.56±0.16 | 11.25±0.08 | 11.32±0.11 | 11.41±0.71 |
| **2** | 6.93±0.37 | 12.01±0.21 | 12.37±0.06 | 12.45±0.32 |
| **3** | 5.42±0.21 | 12.68±0.35 | 12.71±0.13 | 12.86±0.28 |
| **4** | 10.12±0.74 | 19.13±0.24 | 19.32±0.08 | 19.55±0.18 |

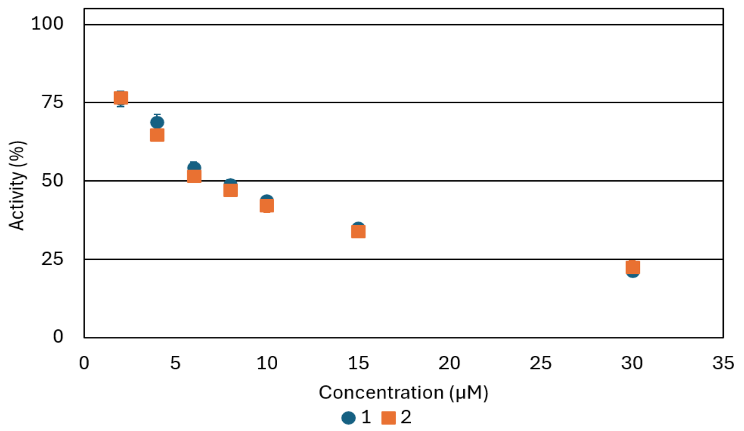


**(A)**

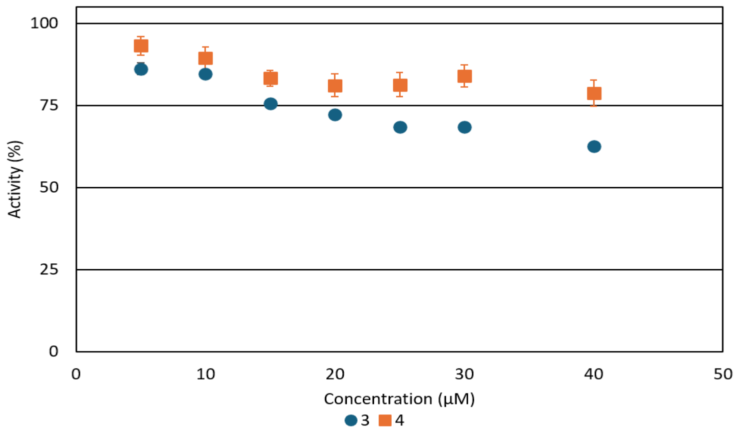


**(B)**

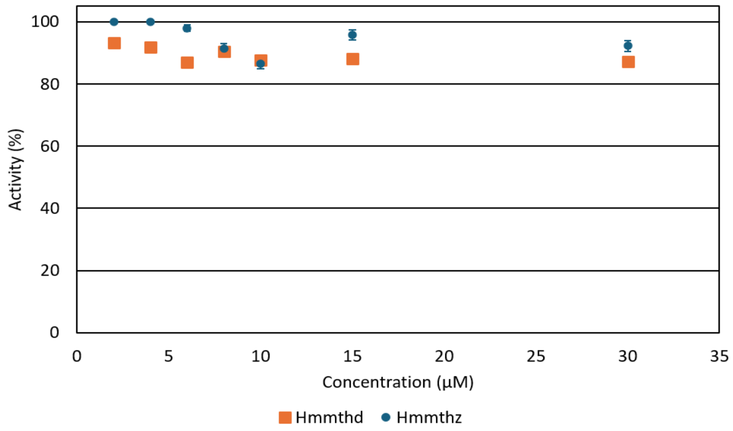


**(C)**

**Figure S30.** Dose-response curves of **1-2** (A), **3-4** (B) and Hmmthd, Hmmthz (C) for the antiproliferative studies towards HeLa cells
