# Supplementary material for: Antimony(III) and Bismuth(III) Halide Complexes Containing Thiazole-and Thiadiazole-Based Mercapto Ligands: Synthesis, Structural Characterization, and Biological Activity
Source: Molecules. 2026 Oct 21;31(20):3783. doi: 10.3390/molecules31203783 (PMC13707501; doi:10.3390/molecules31203783)

checkCIF/PLATON report

Structure factors have been supplied for datablock(s) ibo_122_sm

THIS REPORT IS FOR GUIDANCE ONLY. IF USED AS PART OF A REVIEW PROCEDURE FOR PUBLICATION, IT SHOULD NOT REPLACE THE EXPERTISE OF AN EXPERIENCED CRYSTALLOGRAPHIC REFEREE.

No syntax errors found. CIF dictionary Interpreting this report

Datablock: ibo_122_sm

```
Bond precision:    C-C = 0.0080 A           Wavelength=0.71073

Cell:            a=8.6937(6)        b=10.1525(8)       c=11.1261(10)
                 alpha=67.443(8)    beta=69.827(8)     gamma=69.562(7)
Temperature:     100 K

                  Calculated                    Reported
Volume            824.18(13)                    824.17(14)
Space group       P -1                          P -1
Hall group        -P 1                          -P 1
Moiety formula    C12 H16 Bi2 Br6 N8 S8         C6 H8 Bi Br3 N4 S4
Sum formula       C12 H16 Bi2 Br6 N8 S8         C6 H8 Bi Br3 N4 S4
Mr                1426.17                       713.11
Dx,g cm-3         2.873                         2.874
Z                 1                             2
Mu (mm-1)         18.462                        18.462
F000              648.0                         648.0
F000'             639.97
h,k,lmax          10,12,13                      10,12,13
Nref              3411                          3185
Tmin,Tmax         0.017,0.025                   0.124,1.000
Tmin'             0.000

Correction method= # Reported T Limits: Tmin=0.124 Tmax=1.000
AbsCorr = MULTI-SCAN
```

```
Data completeness= 0.934            Theta(max)= 26.516

                                                       wR2(reflections)=
R(reflections)= 0.0270( 3058)                          0.0677( 3185)
S = 1.128                   Npar= 166
```

The following ALERTS were generated. Each ALERT has the format
test-name_ALERT_alert-type_alert-level.
Click on the hyperlinks for more details of the test.

Alert level C

```
PLAT041_ALERT_1_C Calc. and Reported SumFormula    Strings  Differ     Please Check
              Calc: C12 H16 Bi2 Br6 N8 S8
              Rep.: C6 H8 Bi Br3 N4 S4
PLAT042_ALERT_1_C Calc. and Reported MoietyFormula Strings  Differ     Please Check
              Calc: C12 H16 Bi2 Br6 N8 S8
              Rep.: C6 H8 Bi Br3 N4 S4
PLAT911_ALERT_3_C Missing FCF Refl Between Thmin & STh/L=    0.600          2 Report
                0  1  1,   1  2  1,
PLAT971_ALERT_2_C Check Calcd Resid. Dens.  0.83Ang From Bi1             1.58 eA-3
PLAT971_ALERT_2_C Check Calcd Resid. Dens.  1.11Ang From Bi1             1.51 eA-3
PLAT972_ALERT_2_C Check Calcd Resid. Dens.  0.91Ang From Bi1            -2.15 eA-3
PLAT972_ALERT_2_C Check Calcd Resid. Dens.  0.94Ang From Bi1            -1.98 eA-3
PLAT972_ALERT_2_C Check Calcd Resid. Dens.  0.80Ang From Bi1            -1.53 eA-3
```

Alert level G

```
PLAT007_ALERT_5_G Number of Unrefined Donor-H Atoms .............          2 Report
              H3      H13
PLAT045_ALERT_1_G Calculated and Reported Z Differ by a Factor ...      0.500 Check
PLAT232_ALERT_2_G Hirshfeld Test Diff (M-X)  Bi1      --Br3      .        5.7 s.u.
PLAT232_ALERT_2_G Hirshfeld Test Diff (M-X)  Bi1      --Br3_a    .        8.3 s.u.
PLAT380_ALERT_4_G Incorrectly? Oriented X(sp2)-Methyl Moiety .....       C15' Check
PLAT720_ALERT_4_G Number of Unusual/Non-Standard Labels ..........          3 Note
              H5'A    H5'B    H5'C
PLAT794_ALERT_5_G Tentative Bond Valency for Bi1       (III)     .       3.07 Info
PLAT910_ALERT_3_G Missing FCF Reflection(s) Below Theta(Min)[Deg]=       2.24 Note
                0  0  1,
PLAT912_ALERT_4_G Missing # of FCF Reflections Above STh/L=  0.600        223 Note
PLAT933_ALERT_2_G Number of HKL-OMIT Records in Embedded .res File          1 Note
                0  0  1,
PLAT941_ALERT_3_G Average HKL Measurement Multiplicity ..........         1.8 Low
PLAT969_ALERT_5_G The 'Henn et al.' R-Factor-gap value ..........       2.221 Note
              Predicted wR2: Based on SigI**2  3.05 or SHELX Weight  6.00
PLAT978_ALERT_2_G Number C-C Bonds with Positive Residual Density.          0 Info
PLAT994_ALERT_1_G SHELXL .ins Contains no or MERG 0 Instruction ..          ! Note
```

---

0 **ALERT level A** = Most likely a serious problem - resolve or explain
0 **ALERT level B** = A potentially serious problem, consider carefully
8 **ALERT level C** = Check. Ensure it is not caused by an omission or oversight
14 **ALERT level G** = General information/check it is not something unexpected

```
4 ALERT type 1 CIF construction/syntax error, inconsistent or missing data
9 ALERT type 2 Indicator that the structure model may be wrong or deficient
3 ALERT type 3 Indicator that the structure quality may be low
3 ALERT type 4 Improvement, methodology, query or suggestion
3 ALERT type 5 Informative message, check
```

It is advisable to attempt to resolve as many as possible of the alerts in all categories. Often the minor alerts point to easily fixed oversights, errors and omissions in your CIF or refinement strategy, so attention to these fine details can be worthwhile. It is up to the individual to critically assess their own results and, if necessary, seek expert advice.

PLATON version of 15/01/2026; check.def file version of 02/01/2026

---

duplicate check

No duplication found

Datablock ibo_122_sm - ellipsoid plot

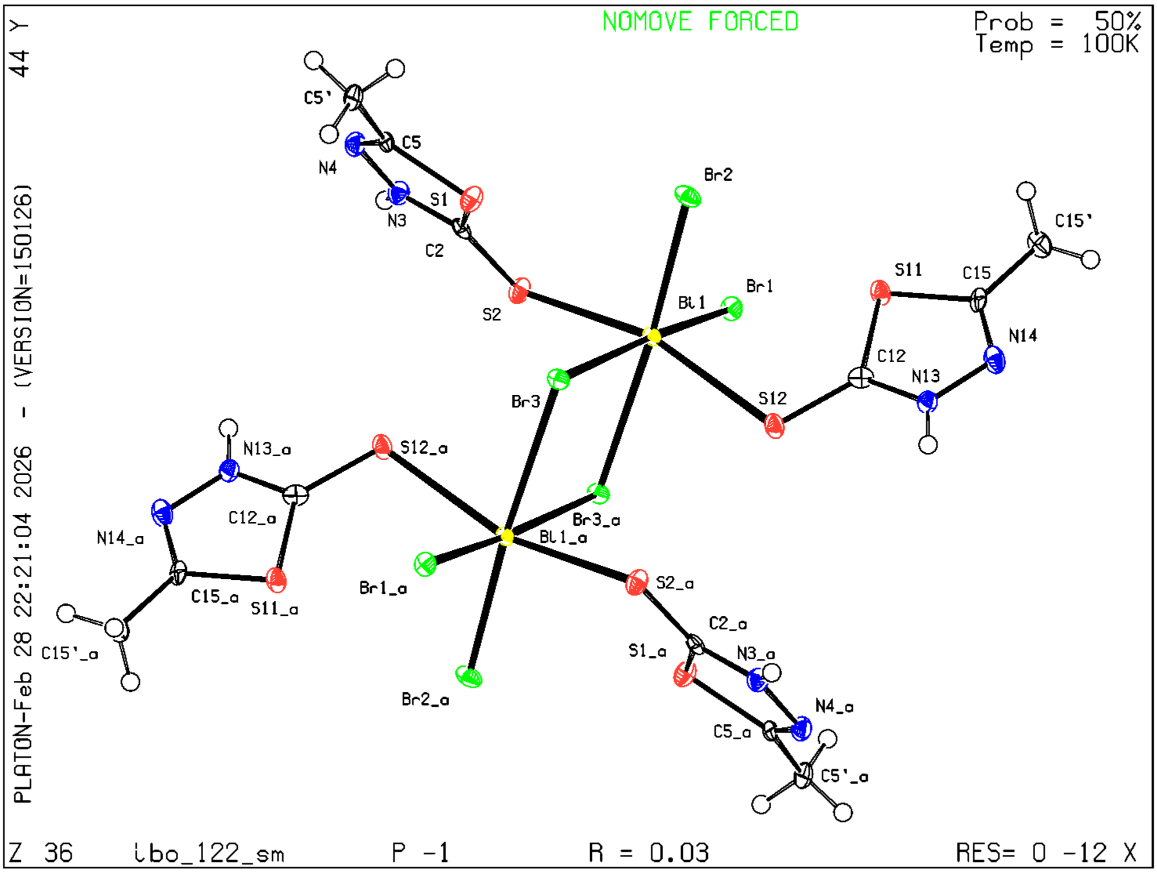

Supplement: Supplementary file 1 [file molecules-31-03783-s001.zip › 2534027.pdf]
