# Supplementary material for: Antimony(III) and Bismuth(III) Halide Complexes Containing Thiazole-and Thiadiazole-Based Mercapto Ligands: Synthesis, Structural Characterization, and Biological Activity
Source: Molecules. 2026 Oct 21;31(20):3783. doi: 10.3390/molecules31203783 (PMC13707501; doi:10.3390/molecules31203783)

checkCIF/PLATON report

Structure factors have been supplied for datablock(s) ibo_144_3

THIS REPORT IS FOR GUIDANCE ONLY. IF USED AS PART OF A REVIEW PROCEDURE FOR PUBLICATION, IT SHOULD NOT REPLACE THE EXPERTISE OF AN EXPERIENCED CRYSTALLOGRAPHIC REFEREE.

No syntax errors found. CIF dictionary Interpreting this report

Datablock: ibo_144_3

Bond precision: C-C = 0.0262 A Wavelength=1.54184

Cell:	a=23.1112(8)	b=8.1067(2)	c=20.9778(7)
	alpha=90	beta=117.003(4)	gamma=90
Temperature:	130 K		

	Calculated	Reported
Volume	3501.8(2)	3501.8(2)
Space group	P 21/c	P 1 21/c 1
Hall group	-P 2ybc	-P 2ybc
Moiety formula	C8 H10 Br2.15 N2 S4 Sb, C8 H10 Br2 N2 S4 Sb, 1.848(Br)	2(C16 H20 Br4.304 N4 S8 Sb2), 3.696(Br1), 2(C8 H10 Br2 N2 S4 Sb
Sum formula	C16 H20 Br6 N4 S8 Sb2	C32 H40 Br12 N8 S16 Sb4
Mr	1247.76	2495.60
Dx,g cm-3	2.367	2.367
Z	4	2
Mu (mm-1)	24.868	24.867
F000	2336.0	2336.0
F000’	2333.31	
h,k,lmax	27,9,25	27,9,25
Nref	6408	6397
Tmin,Tmax	0.381,0.440	0.355,1.000
Tmin’	0.251	

```
Correction method= # Reported T Limits: Tmin=0.355 Tmax=1.000
AbsCorr = MULTI-SCAN

Data completeness= 0.998            Theta(max)= 68.277

R(reflections)= 0.0609( 5005)                       wR2(reflections)=
                                                    0.1660( 6397)
S = 1.031                   Npar= 386
```

The following ALERTS were generated. Each ALERT has the format
test-name_ALERT_alert-type_alert-level.
Click on the hyperlinks for more details of the test.

Alert level B

```
PLAT342_ALERT_3_B Low Bond Precision on  C-C Bonds ..............    0.02625 Ang.
```

Alert level C

```
PLAT041_ALERT_1_C Calc. and Reported SumFormula    Strings  Differ     Please Check
              Calc: C16 H20 Br6 N4 S8 Sb2
              Rep.: C32 H40 Br12 N8 S16 Sb4
PLAT042_ALERT_1_C Calc. and Reported MoietyFormula Strings  Differ     Please Check
              Calc: C8 H10 Br2.15 N2 S4 Sb, C8 H10 Br2 N2 S4 Sb, 1
                    .848(Br)
              Rep.: 2(C16 H20 Br4.304 N4 S8 Sb2), 3.696(Br1), 2(C8
                     H10 Br2 N2 S4 Sb)
PLAT213_ALERT_2_C Atom S22A            has ADP max/min Ratio .....        3.1 prolat
PLAT234_ALERT_4_C Large Hirshfeld Difference S21      --C22      .       0.18 Ang.
PLAT234_ALERT_4_C Large Hirshfeld Difference S21      --C25      .       0.16 Ang.
PLAT234_ALERT_4_C Large Hirshfeld Difference S31      --C35      .       0.18 Ang.
PLAT234_ALERT_4_C Large Hirshfeld Difference N23      --C22      .       0.18 Ang.
PLAT234_ALERT_4_C Large Hirshfeld Difference C24      --C25      .       0.17 Ang.
PLAT234_ALERT_4_C Large Hirshfeld Difference N3       --C4       .       0.20 Ang.
PLAT234_ALERT_4_C Large Hirshfeld Difference N13      --C12      .       0.17 Ang.
PLAT241_ALERT_2_C High  MainResAtom  Ueq as Compared to Neighbours        S31 Check
PLAT241_ALERT_2_C High  MainResAtom  Ueq as Compared to Neighbours         S2 Check
PLAT241_ALERT_2_C High  MainResAtom  Ueq as Compared to Neighbours        S12 Check
PLAT242_ALERT_2_C Low   MainResAtom  Ueq as Compared to Neighbours        Sb1 Check
PLAT242_ALERT_2_C Low   MainResAtom  Ueq as Compared to Neighbours        C12 Check
PLAT250_ALERT_2_C Large U3/U1 Ratio for <U(i,j)> Tensor(Resd    1)        2.4 Note
PLAT303_ALERT_2_C Full Occupancy Atom H24A      with # Connections       1.17 Check
PLAT303_ALERT_2_C Full Occupancy Atom H33       with # Connections       1.15 Check
PLAT906_ALERT_3_C Large K Value in the Analysis of Variance ......      2.645 Check
PLAT911_ALERT_3_C Missing FCF Refl Between Thmin & STh/L=    0.600         10 Report
                2  0  0,  -2  0  2,   0  0  2, -10  9  3,  -6  0  4,   2  0  4,
              -14  8  6, -10  0  6,   4  0  6,  20  0  6,
```

Alert level G

```
FORMU01_ALERT_1_G  There is a discrepancy between the atom counts in the
            _chemical_formula_sum and _chemical_formula_moiety. This is
            usually due to the moiety formula being in the wrong format.
```

```
            Atom count from _chemical_formula_sum:   C32 H40 Br12 N8 S16 Sb4
            Atom count from _chemical_formula_moiety:C48 H60 Br16.30400 N12 S24 Sb
PLAT002_ALERT_2_G Number of Distance or Angle Restraints on AtSite          6 Note
PLAT003_ALERT_2_G Number of Uiso or U(i,j)  Restrained non-H-Atoms          1 Report
PLAT004_ALERT_5_G Polymeric Structure Found with Maximum Dimension          1 Info
PLAT007_ALERT_5_G Number of Unrefined Donor-H Atoms .............           4 Report
              H23     H33     H3      H13
PLAT045_ALERT_1_G Calculated and Reported Z Differ by a Factor ...          2 Check
PLAT176_ALERT_4_G The CIF-Embedded .res File Contains SADI Records          5 Report
PLAT186_ALERT_4_G The CIF-Embedded .res File Contains ISOR Records          1 Report
PLAT191_ALERT_3_G A Non-default SADI Restraint Value has been used     0.0400 Report
PLAT191_ALERT_3_G A Non-default SADI Restraint Value has been used     0.0400 Report
PLAT191_ALERT_3_G A Non-default SADI Restraint Value has been used     0.0400 Report
PLAT301_ALERT_3_G Main Residue  Disorder .............(Resd    1)        30% Note
PLAT302_ALERT_4_G Anion/Solvent/Minor-Residue Disorder (Resd    4)       100% Note
PLAT720_ALERT_4_G Number of Unusual/Non-Standard Labels ..........          3 Note
              H4'A    H4'B    H4'C
PLAT780_ALERT_1_G Coordinates do not Form a Properly Connected Set     Please Do !
PLAT794_ALERT_5_G Tentative Bond Valency for Sb1       (III)     .       2.92 Info
PLAT860_ALERT_3_G Number of Least-Squares Restraints ............           9 Note
PLAT870_ALERT_4_G ALERTS Related to Twinning Effects Suppressed ..          ! Info
PLAT910_ALERT_3_G Missing FCF Reflection(s) Below Theta(Min)[Deg]=       4.21 Note
                1  0  0,
PLAT913_ALERT_3_G Missing # of Very Strong Reflections in FCF ....          2 Note
              -10  0  6,   4  0  6,
PLAT931_ALERT_5_G CIFcalcFCF  Twin Law ( 1 0-2)         Est.d BASF       0.52 Check
PLAT933_ALERT_2_G Number of HKL-OMIT Records in Embedded .res File          3 Note
               -6  0  4,   0  0  2,   2  0  0,
PLAT941_ALERT_3_G Average HKL Measurement Multiplicity ..........         1.0 Low
PLAT969_ALERT_5_G The 'Henn et al.' R-Factor-gap value ..........       1.298 Note
              Predicted wR2: Based on SigI**2 12.79 or SHELX Weight 16.10 Note
PLAT994_ALERT_1_G SHELXL .ins with Im-/Explicit MERG 0 Instruction          ! Note
```

```
   0 ALERT level A = Most likely a serious problem - resolve or explain
   1 ALERT level B = A potentially serious problem, consider carefully
  20 ALERT level C = Check. Ensure it is not caused by an omission or oversight
  25 ALERT level G = General information/check it is not something unexpected

   6 ALERT type 1 CIF construction/syntax error, inconsistent or missing data
  12 ALERT type 2 Indicator that the structure model may be wrong or deficient
  11 ALERT type 3 Indicator that the structure quality may be low
  12 ALERT type 4 Improvement, methodology, query or suggestion
   5 ALERT type 5 Informative message, check
```

It is advisable to attempt to resolve as many as possible of the alerts in all categories. Often the minor alerts point to easily fixed oversights, errors and omissions in your CIF or refinement strategy, so attention to these fine details can be worthwhile. It is up to the individual to critically assess their own results and, if necessary, seek expert advice.

PLATON version of 20/06/2026; check.def file version of 19/06/2026

Datablock ibo_144_3 - ellipsoid plot

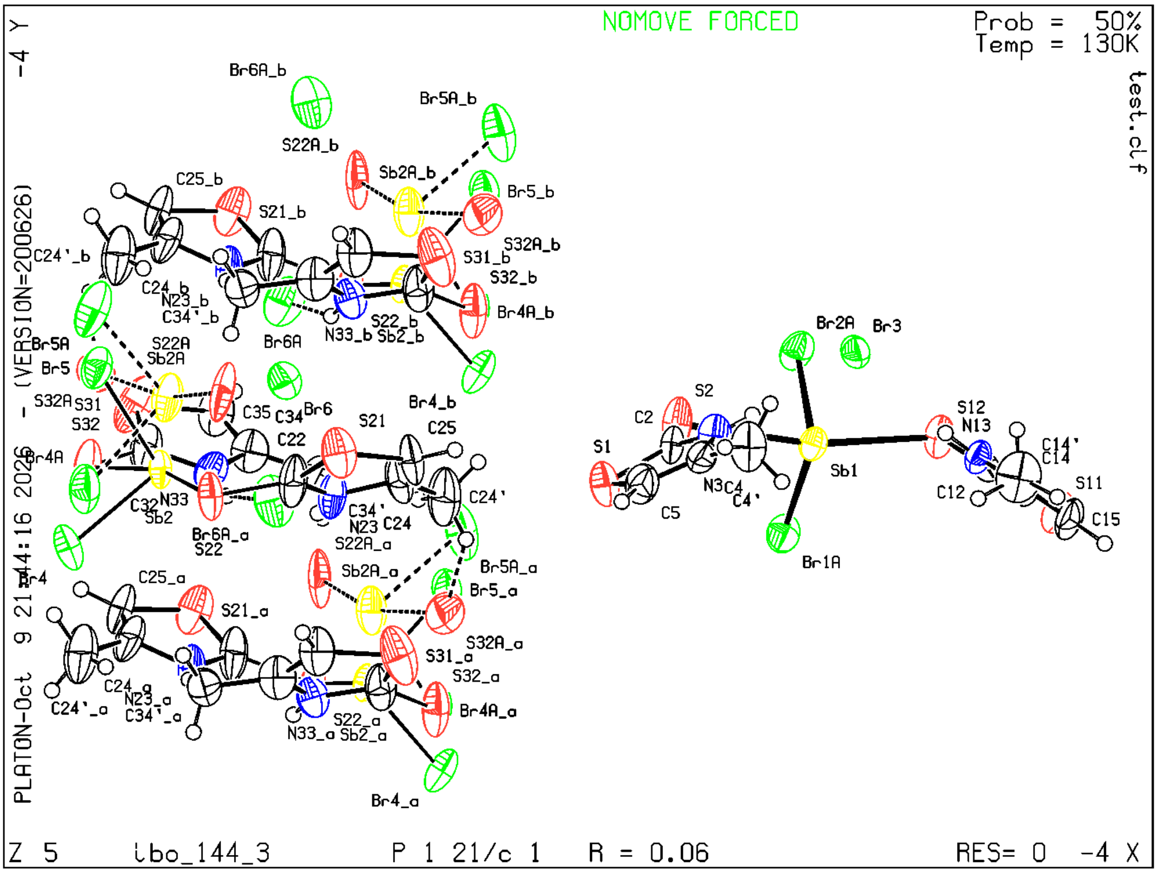

Supplement: Supplementary file 1 [file molecules-31-03783-s001.zip › 2534028.pdf]
