# Supplementary material for: Antimony(III) and Bismuth(III) Halide Complexes Containing Thiazole-and Thiadiazole-Based Mercapto Ligands: Synthesis, Structural Characterization, and Biological Activity
Source: Molecules. 2026 Oct 21;31(20):3783. doi: 10.3390/molecules31203783 (PMC13707501; doi:10.3390/molecules31203783)

# checkCIF/PLATON report

Structure factors have been supplied for datablock(s) ibo_141_c_2c

THIS REPORT IS FOR GUIDANCE ONLY. IF USED AS PART OF A REVIEW PROCEDURE FOR PUBLICATION, IT SHOULD NOT REPLACE THE EXPERTISE OF AN EXPERIENCED CRYSTALLOGRAPHIC REFEREE.

No syntax errors found. CIF dictionary Interpreting this report

## Datablock: ibo_141_c_2c

---

Bond precision: C-C = 0.0130 A Wavelength=0.71073

| | | | |
|---|---|---|---|
| Cell: | a=9.6910(14) | b=22.303(4) | c=8.0863(13) |
| | alpha=90 | beta=104.923(5) | gamma=90 |
| Temperature: | 273 K | | |

| | Calculated | Reported |
|---|---|---|
| Volume | 1688.8(5) | 1688.9(5) |
| Space group | C 2/c | C 1 2/c 1 |
| Hall group | -C 2yc | -C 2yc |
| Moiety formula | C8 H10 Bi Cl3 N2 S4 | C8 H10 Bi Cl3 N2 S4 |
| Sum formula | C8 H10 Bi Cl3 N2 S4 | C8 H10 Bi Cl3 N2 S4 |
| Mr | 577.75 | 577.75 |
| Dx,g cm-3 | 2.272 | 2.272 |
| Z | 4 | 4 |
| Mu (mm-1) | 11.393 | 11.392 |
| F000 | 1080.0 | 1080.0 |
| F000' | 1068.20 | |
| h,k,lmax | 11,26,9 | 11,26,9 |
| Nref | 1549 | 1545 |
| Tmin,Tmax | 0.585,0.796 | 0.242,0.745 |
| Tmin' | 0.317 | |

Correction method= # Reported T Limits: Tmin=0.242 Tmax=0.745
AbsCorr = MULTI-SCAN

Data completeness= 0.997          Theta(max)= 25.350

R(reflections)= 0.0352( 1351)          wR2(reflections)= 0.0754( 1545)

S = 1.042          Npar= 85

---

The following ALERTS were generated. Each ALERT has the format
**test-name_ALERT_alert-type_alert-level**.
Click on the hyperlinks for more details of the test.

---

## Alert level C

```
RINTA01_ALERT_3_C  The value of Rint is greater than 0.12
            Rint given   0.179
PLAT242_ALERT_2_C Low   MainResAtom  Ueq as Compared to Neighbours        Bi1 Check
PLAT342_ALERT_3_C Low Bond Precision on  C-C Bonds ...............      0.013 Ang.
PLAT911_ALERT_3_C Missing FCF Refl Between Thmin & STh/L=    0.600          3 Report
                5 13  0,  -9  5  2, -10  6  2,
```

---

## Alert level G

```
PLAT004_ALERT_5_G Polymeric Structure Found with Maximum Dimension          1 Info
PLAT007_ALERT_5_G Number of Unrefined Donor-H Atoms .............          1 Report
              H4
PLAT020_ALERT_3_G The Reported Value of Rint is Greater Than  0.12      0.179 Report
PLAT199_ALERT_1_G Reported _cell_measurement_temperature ..... (K)        273 Check
PLAT200_ALERT_1_G Reported   _diffrn_ambient_temperature ..... (K)        273 Check
PLAT720_ALERT_4_G Number of Unusual/Non-Standard Labels .........          3 Note
              H3'A    H3'B    H3'C
PLAT794_ALERT_5_G Tentative Bond Valency for Bi1       (III)     .       3.28 Info
PLAT910_ALERT_3_G Missing FCF Reflection(s) Below Theta(Min)[Deg]=       2.36 Note
                0  2  0,
PLAT933_ALERT_2_G Number of HKL-OMIT Records in Embedded .res File          3 Note
              -10  6  2,  -9  5  2,   5 13  0,
PLAT969_ALERT_5_G The 'Henn et al.' R-Factor-gap value ..........      1.043 Note
              Predicted wR2: Based on SigI**2  7.23 or SHELX Weight  7.23 Note
PLAT978_ALERT_2_G Number C-C Bonds with Positive Residual Density.          0 Info
```

---

0 **ALERT level A** = Most likely a serious problem - resolve or explain
0 **ALERT level B** = A potentially serious problem, consider carefully
4 **ALERT level C** = Check. Ensure it is not caused by an omission or oversight
11 **ALERT level G** = General information/check it is not something unexpected

2 ALERT type 1 CIF construction/syntax error, inconsistent or missing data
3 ALERT type 2 Indicator that the structure model may be wrong or deficient
5 ALERT type 3 Indicator that the structure quality may be low
1 ALERT type 4 Improvement, methodology, query or suggestion
4 ALERT type 5 Informative message, check

---

---

It is advisable to attempt to resolve as many as possible of the alerts in all categories. Often the minor alerts point to easily fixed oversights, errors and omissions in your CIF or refinement strategy, so attention to these fine details can be worthwhile. It is up to the individual to critically assess their own results and, if necessary, seek expert advice.

---

**PLATON version of 20/06/2026; check.def file version of 19/06/2026**

---

# duplicate check

**No duplication found**

---

**Datablock ibo_141_c_2c** - ellipsoid plot

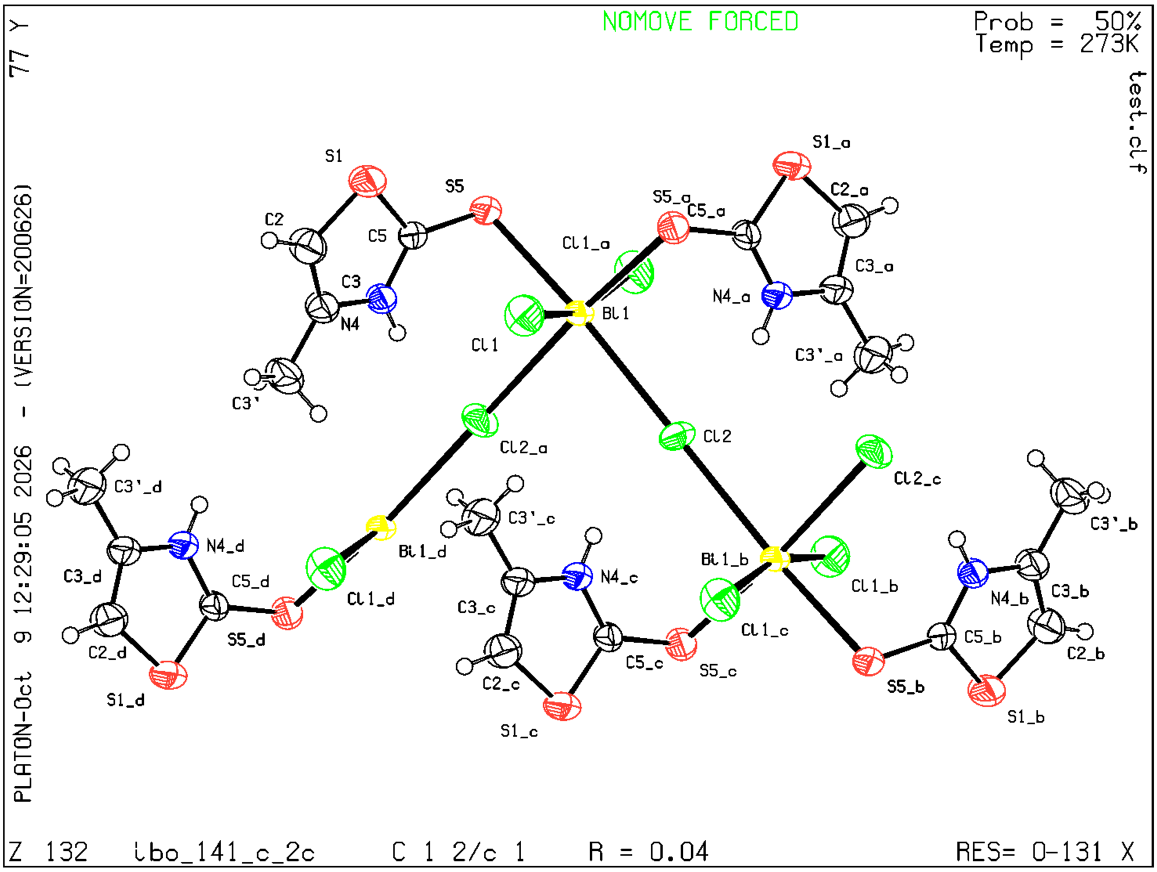

Supplement: Supplementary file 1 [file molecules-31-03783-s001.zip › 2534029.pdf]
